# Antibody-Drug Conjugates in Solid Tumor Oncology and the Frontier of Precision Immunosuppression: A Mechanistic, Translational, and Clinical Review

**DOI:** 10.3390/ijms27125196

**Published:** 2026-06-09

**Authors:** Ibraheem Masoud, Nada Saed Homod Al Shaer, Ahmad Masoud, Ahmad Al Jandali, Abdulrahman Aldahash, Abdullah Jabri, Mohamed Alsharif, Fareeha Arshad, Itika Arora, Mohammed Imran Khan, Ahmed Yaqinuddin

**Affiliations:** 1 College of Medicine, Alfaisal University, Riyadh 11533, Saudi Arabia; imasoud@alfaisal.edu (I.M.); nalshaer@alfaisal.edu (N.S.H.A.S.); amasoud@alfaisal.edu (A.M.); aaljandali@alfaisal.edu (A.A.J.); amaldahash@alfaisal.edu (A.A.); ajabri@alfaisal.edu (A.J.); moalsharif@alfaisal.edu (M.A.); farshad@alfaisal.edu (F.A.); iarora@alfaisal.edu (I.A.); 2 Research Center, King Faisal Specialist Hospital and Research Center, Jeddah 21499, Saudi Arabia; mikhan@kfshrc.edu.sa

**Keywords:** antibody-drug conjugate, trastuzumab deruxtecan, enfortumab vedotin, tumor heterogeneity, bystander effect, linker-payload axis, solid tumor, precision immunosuppression

## Abstract

Antibody-drug conjugates (ADCs) have transitioned from clinically marginal agents into a defining therapeutic class for solid tumor oncology. In DESTINY-Breast03, trastuzumab deruxtecan achieved a four-fold progression-free survival advantage over trastuzumab emtansine, attributable not to antibody engineering but to the linker-payload axis: a cleavable peptide linker and a topoisomerase I payload with bystander activity. Sacituzumab govitecan extends the same logic to Trop-2-positive disease via extracellular payload release, and the framework now spans breast, urothelial, gynecologic, lung, gastric, and colorectal cancers, with enfortumab vedotin plus pembrolizumab displacing platinum chemotherapy as first-line therapy for urothelial cancer in EV-302 (median overall survival 31.5 versus 16.1 months). This review synthesizes ADC biology along three analytical axes. The mechanistic axis links each linker-payload-DAR configuration to a specific tumor-biology barrier: vascular limitation, which delivers approximately 0.1% of the administered dose to tumor tissue; the binding-site barrier, which concentrates exposure at the perivascular margin; and antigen mosaicism, which defeats internalization-dependent killing. The translational axis examines resistance as a coordinated failure across antigen modulation, trafficking, efflux, apoptotic execution, and lysosomal processing. The clinical axis traces the platform’s migration toward earlier-line and curative-intent settings. We close by examining whether the ADC delivery architecture translates to precision immunosuppression in autoimmune disease, where the glucocorticoid receptor modulator ADC ABBV-154 met placebo-controlled efficacy endpoints in rheumatoid arthritis but was discontinued because its benefit-risk profile did not differentiate it from existing biologic therapies.

## 1. Introduction

**Figure 1:**
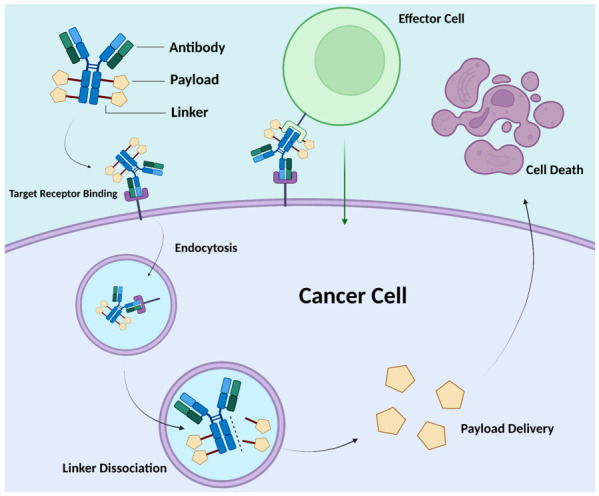
Antibody-drug conjugate structure and mechanisms of action. This schematic represents the basic structure of antibody-drug conjugates (ADCs), in which an antibody is loaded with a cytotoxic payload attached via a cleavable linker. Antibodies bind specifically to receptors expressed on malignant cells and are then endocytosed. Once inside the target cell, the linker dissociates, allowing the cytotoxic payload to take effect. ADC antibodies also trigger host effector cells to elicit an immune response against malignant cells.

The conceptual foundation of antibody-drug conjugates originates in Paul Ehrlich’s early 1900s “magic bullet” hypothesis. Yet nearly a century of iterative engineering separated this vision from its first clinical realization, reflecting not a failure of principle but a persistent mismatch between ADC chemistry and the biological requirements of tumor-selective cytotoxic delivery [1]. First-generation ADCs carried conventional chemotherapeutic payloads, including vinca alkaloids, methotrexate, and doxorubicin, which proved no more potent than their unconjugated counterparts, rendering the delivered cytotoxic dose insufficient to elicit meaningful antitumor activity [1]. The approval of gemtuzumab ozogamicin in 2000 as the first FDA-authorized ADC, and its subsequent voluntary withdrawal in 2010, exemplified this class of failure at higher resolution: the conjugate’s linker could not maintain payload attachment during systemic circulation, and the resulting premature release of calicheamicin produced toxicity that outweighed any therapeutic gain. The drug was reapproved by the FDA in 2017 at a fractionated lower-dose schedule following improved benefit-risk data from the ALFA-0701 trial [2]. Second-generation constructs corrected this specific deficiency by adopting humanized antibodies and redesigning linkage and conjugation chemistries to achieve DAR values consistently near 3–4, thereby enabling potent microtubule inhibitors, MMAE in brentuximab vedotin and DM1 in trastuzumab emtansine, to reach clinical use [3]. The third generation of ADCs, defined by the regulatory approval of trastuzumab deruxtecan in late 2019 following the DESTINY-Breast01 phase II data, resolved this constraint by incorporating topoisomerase I payloads, including DXd and SN-38, which inhibit topoisomerase I and generate single-strand DNA breaks that are converted into cytotoxic double-strand breaks during replication. Their bystander activity also enables destruction of neighboring tumor cells that do not themselves express the target antigen, a capability that no earlier ADC generation possessed [3,4] (Figure 1).

The generational progression described above unfolded against a set of biological constraints unique to solid tumors, in which drug delivery, intratumoral distribution, and tumor-cell exposure are jointly restricted by barriers that do not arise in the same form in hematologic settings, where ADCs first proved effective. The scale of this problem is quantifiable: only approximately 0.1% of an administered ADC dose reaches the tumor compartment [1,5]. Within the tumor itself, the physical bulk of the intact ADC, abnormal vasculature, hypoxic gradients, and elevated interstitial pressure result in poor penetration, constituting an early resistance mechanism that operates independently of antigen expression [6], and this is further compounded by heterogeneous expression of target antigens across the tumor mass [3]. Layered onto this physical exclusion is a kinetic constraint specific to ADC pharmacology, the binding-site barrier (BSB), in which rapid perivascular antigen binding outpaces diffusion and leaves distal cancer cells untargeted; this barrier is detailed in Section 3.2 [7,8]. The BSB poses a greater challenge for ADCs than for unconjugated antibodies, as payload-driven dose-limiting toxicities force ADC administration at relatively lower doses, and tumors with high antigen density paradoxically suffer most, as rapid target engagement at the perivascular margin depletes the conjugate before it can reach deeper tissue [8]. At the cellular level, a third constraint compounds the first two: antigen heterogeneity generates mosaic populations of antigen-positive and antigen-negative cells within the same lesion, limiting uniform cytotoxic exposure for ADC platforms that depend on receptor-mediated internalization to deliver their payload [3,9]. These three barriers do not operate in isolation. Aberrant vasculature concentrates ADC at the perivascular front, where BSB kinetics further restrict redistribution into the tumor core. Among the narrow subset of cells that receive therapeutic exposure, antigen mosaicism reduces the fraction susceptible to internalization-dependent killing. This mechanistic convergence is what made solid tumors categorically resistant to the receptor-targeting design logic that had succeeded in hematologic malignancies.

The resolution to the compounding delivery, penetration, and heterogeneity constraints described above emerged not from antibody re-engineering but from the linker-payload axis. T-DXd and T-DM1 share an identical HER2-targeting antibody backbone but differ in their conjugated cytotoxic agent and linker chemistry [10,11]: T-DM1 pairs its antibody to a microtubule-disrupting agent through a bond that resists cleavage in circulation [12]. At the same time, T-DXd uses a peptide-based linkage designed to remain intact in plasma but to undergo selective scission once inside malignant cells, releasing a topoisomerase I-targeting cytotoxin [13,14]. In the DESTINY-Breast03 head-to-head comparison, this linker-payload difference resulted in a fourfold difference in PFS, with T-DXd achieving a median of 29.0 months versus 7.2 months for T-DM1 [15]. The mechanism underlying this disparity is the bystander effect: T-DXd’s released payload crosses into adjacent cells regardless of whether they carry HER2 on their surface, which means that mixed tumor populations containing both HER2-expressing and HER2-negative cells can be killed, a property that made low-level and heterogeneous HER2 expression targetable for the first time [16,17]. T-DXd’s reach has since been extended to most patients with HR-positive, HER2-negative metastatic breast cancer through the DESTINY-Breast06 trial, which included the newly defined HER2-ultralow subgroup [17]. A mechanistically distinct yet convergent approach is exemplified by sacituzumab govitecan, in which the cytotoxic agent SN-38 reaches tumor tissue not through a single delivery route but through a combination of whole-conjugate uptake into target cells and local liberation of free drug outside the cell, with both pathways contributing simultaneously [18]. Clinical data show that this agent produces better outcomes than standard single-agent chemotherapy with a tolerable side-effect burden and suggest it may retain activity even when tumors have stopped responding to chemotherapeutic agents given at earlier stages of treatment [19]. Together, these two agents show that the delivery barriers above are addressable through linker-payload design rather than antibody re-engineering.

The clinical validation of these design principles has been rapid, but the broadening application of ADCs has simultaneously surfaced unresolved problems at every level of the platform, from the molecular design of the conjugate and the choice of target antigen, through resistance and toxicity, to combination strategy and the challenge of making these agents globally accessible [3,20]. At the pharmacologic level, progress requires the ability to distinguish on-target from off-target mechanisms of toxicity, and to map how linker design and conjugation approach determine where the ADC and payload distribute across the body, steps that are necessary to produce agents with improved safety, greater potency, and activity in tumors that have acquired resistance to currently available ADCs [4]. This review is organized around three analytical axes: a mechanistic axis that links each linker-payload-DAR configuration to a specific tumor biology barrier; a translational axis that examines resistance pathways, multi-omics data, and biomarker-guided patient selection; and a clinical axis that surveys evidence by tumor type and target class across breast (HER2-spectrum, Trop-2, HER3), urothelial (Nectin-4), gynecologic (tissue-factor, folate receptor alpha), non-small cell lung (HER2-mutant, TROP2, c-MET), and gastric and colorectal HER2-positive disease. Hematologic malignancies, manufacturing process chemistry, and pharmacoeconomics fall outside the scope of this work. ADC design is evolving beyond agents built solely to deliver cytotoxic payloads and toward platforms that integrate multiple therapeutic functions [3]. We contend that extending these principles into non-oncologic domains, particularly selective immunomodulation in autoimmune disease, represents a logical but largely untested frontier.

## 2. Evolution of ADC Design: From First to Next Generation

### 2.1. Antibody Selection

The challenge of finding effective antibodies to guide delivery of cytotoxic payloads was first overcome in the 1990s, when the earliest reports of successful ADC designs utilizing murine-derived antibodies were published [21,22]. The murine-derived antibodies initially used, though a step forward, elicited an immune response in recipients, resulting in the formation of host Anti-Drug Antibodies (ADAs) against the antibodies [23]. Recent designs have overcome this challenge by implementing humanized or fully human immunoglobulin G (IgG) scaffolds. Although multiple isotypes are used, IgG1 is preferred in most commercially available ADCs because the naked antibody isotype has a long serum half-life of approximately 21 days, mediated by FcRn recycling, and because IgG1 engages immune effector functions, notably antibody-dependent cellular cytotoxicity (ADCC), through Fcγ receptors. The intact ADC molecule, however, exhibits a substantially shorter effective half-life than the unconjugated antibody, reflecting conjugation-related clearance and payload-linked catabolism, which vary with linker chemistry and drug-to-antibody ratio [24,25]. Isotype selection also impacts the stability of the conjugation sites; IgG1 provides a balance of flexibility and structural integrity, and IgG4 has been shown to have a reduced propensity to trigger complement-dependent cytotoxicity (CDC), which may be preferable when minimizing on-target, off-tumor inflammation is the priority [26]. Additionally, recent advances have been made through Fc-region engineering, in which silencing the Fc region of the chosen antibody has been reported to reduce non-specific uptake by healthy immune cells expressing Fc receptors, thereby widening the therapeutic window [27].

The concept of antigen density is also being re-evaluated. First-generation designs typically required relatively high or homogeneous target expression, though even CD33 in AML exhibited meaningful variability in expression across blasts. However, next-generation ADCs like Trastuzumab deruxtecan have demonstrated that high payload potency and favorable bystander effects can achieve clinical responses in “low-antigen” environments [28], which allows clinicians to consider ADCs for a broader range of patients, moving beyond binary “positive/negative” expression metrics [29,30].

Internalization kinetics represent the “gatekeeper” of intracellular payload delivery. The rate at which an antibody–antigen complex is endocytosed determines the effective concentration of the drug within the lysosome. Research indicates that receptors with rapid turnover or those that trigger clathrin-mediated endocytosis provide superior delivery vehicles [31,32]. Conversely, receptors that recycle the ADC back to the cell surface can sequester the payload, leading to therapeutic failure and acquired resistance [33]. High-affinity antibodies can be trapped at the perivascular margin (the binding-site barrier; see Section 3.2), so modern designs target a moderate-affinity “sweet spot” that balances specificity with deep, homogeneous tissue distribution [34,35].

### 2.2. Linker Chemistry

Linkers serve as the critical “safety catch” of the ADC, ensuring that the cytotoxic payload remains sequestered until it reaches the malignant cell, where it is ideally released from the antibody-drug conjugate complex. The shift from acid-labile linkers (such as the hydrazone linker in Gemtuzumab ozogamicin) to enzyme-cleavable linkers represents a major improvement in safety. Acid-labile linkers are prone to premature hydrolysis at the physiologically neutral pH of systemic circulation (7.35–7.45), where retained ester or hydrazone bonds undergo slow but cumulative cleavage that releases the payload into plasma and generates off-target toxicity; selective cleavage was designed to occur at the lower endosomal and lysosomal pH (≤5.0–6.5) reached only after target-cell internalization [36,37]. Modern dipeptide linkers, such as valine-citrulline (Val-Cit), are specifically cleaved by lysosomal proteases like Cathepsin B, thereby restricting release to the intracellular compartment [38,39].

The pharmacological trend toward non-cleavable linkers (e.g., the SMCC linker in T-DM1) was initially favored for its superior plasma stability. These linkers require that the entire antibody be degraded in the lysosome, resulting in a charged, amino acid-linked payload [40,41]. While this virtually eliminates systemic leakage, the resulting metabolite cannot cross cell membranes. Clinically, this manifests as non-cleavable linkers proving highly effective in homogeneous tumors but failing in heterogeneous environments where the “bystander effect” is required [12,42]. A significant biochemical challenge in linker design is the retro-Michael reaction, in which the payload detaches from the antibody and transfers to serum albumin. This phenomenon explains why earlier ADC designs often exhibited “payload-related” toxicities in the liver and bone marrow [43]. Next-generation stabilized linkers utilize ring-opened thiosuccinimides or self-stabilizing maleimides to prevent this exchange, significantly increasing the maximum tolerated dose (MTD) in clinical trials [44].

The hydrophilicity of the linker is also a key driver of contemporary research. Hydrophobic linkers often cause the entire ADC to aggregate, which triggers rapid clearance by the liver’s Kupffer cells [45]. By incorporating polyethylene glycol (PEG) spacers or other hydrophilic moieties, the ADC remains in circulation longer, thereby increasing its pharmacokinetic half-life and ensuring that more of the drug reaches the tumor [43,46].

Ultimately, the trend is moving toward “tunable” release kinetics, often involving specifically engineering the linker to respond to the unique enzymatic signature of a specific tumor type. For instance, linkers sensitive to matrix metalloproteinases (MMPs) can release the payload in the tumor stroma rather than inside the cell, a strategy that has been reported to be particularly useful for overcoming the limitations of poor antibody internalization [29,33,39].

### 2.3. Payloads

The development of payloads has transitioned from standard chemotherapeutics to ultra-potent cytotoxics with IC50 values in the picomolar range. Initial attempts with doxorubicin failed because the antibody could not deliver enough molecules to reach a lethal cytotoxic threshold [1]. In contrast, modern ADCs have been designed to utilize microtubule inhibitors (e.g., MMAE, DM1) or DNA-damaging agents (e.g., DXd, SN-38). Microtubule inhibitors block tubulin polymerization and arrest cells at the metaphase-anaphase transition by activating the spindle assembly checkpoint, an effect that requires active cell division [47,48]. DXd is also highly membrane-permeable, enabling the bystander effect that underlies its activity in HER2-low breast cancer [28,47].

Mechanism-based toxicities are a critical consideration for physicians; Auristatin payloads (e.g., MMAE) are frequently associated with peripheral neuropathy due to their effects on axonal microtubules, whereas topoisomerase inhibitors are more likely to cause gastrointestinal toxicity or interstitial lung disease (ILD) [29]. Understanding the payload’s “warhead” mechanism allows clinicians to predict and preemptively manage these side effects based on the specific drug being administered [33,48]. Resistance to payloads is increasingly linked to upregulation of efflux pumps expressed in target tumors, such as P-glycoprotein (MDR1). When the payload is released intracellularly, efflux pumps actively eject the toxin before it reaches its target [33]. Next-generation payloads are being engineered to be poor substrates for these pumps, effectively bypassing this common mode of chemotherapy resistance [43,48].

Recent advancements in non-cytotoxic payloads have also been reported. Immuno-stimulating payloads, such as STING (Stimulator of Interferon Genes) agonists, are being conjugated to antibodies to “re-prime” the immune system rather than directly killing the cell. This strategy aims to turn “cold” tumors “hot” by activating local dendritic cells and macrophages, potentially creating a synergistic effect with existing checkpoint inhibitors [49].

### 2.4. Drug-to-Antibody Ratio (DAR)

The DAR is a pivotal metric that determines the ADC’s payload density. Historically, a DAR of 3–4 was considered the absolute ceiling. In first-generation designs, increasing the number of payloads beyond 4 led to rapid hepatic clearance and drug aggregation due to the increased hydrophobicity of the complex [50]. Clinically, this meant that higher loads did not translate directly into greater efficacy but rather increased liver toxicity and reduced half-life [46,51]. However, the advent of hydrophilic linker technology and site-specific conjugation has surpassed the DAR of 4 limit. Newer agents such as T-DXd achieve a DAR of approximately 8, with substantially improved homogeneity compared with earlier random conjugation approaches, without sacrificing pharmacokinetic stability [13,47]. This high DAR is essential for targeting tumors with low antigen expression; since fewer antibodies bind to the cell, each binding event must deliver a much larger lethal dose to ensure cell death [28,29].

The impact of DAR heterogeneity is a major focus of current regulatory and clinical rigor. Random conjugation results in a mixture of “empty” antibodies (DAR 0), which compete for target binding without delivering the drug, and “overloaded” antibodies (DAR > 6), which are cleared too fast. Achieving a homogenous DAR where nearly every molecule carries the same amount of payload ensures more predictable dosing, a cleaner safety profile, and improved therapeutic indices [43,51,52]. Biochemically, the placement of the payload on the antibody (the conjugation site) is as important as the number of payloads. Site-specific conjugation ensures that the payload is attached at a location that does not interfere with the antibody’s ability to bind to its receptor or its neonatal Fc receptor (FcRn), which protects it from degradation [52,53]. This precision engineering is the hallmark of “Third Generation” ADCs [33,36].

The future direction of DAR design is patient-tailored loading. For hematological malignancies where targets are abundant and cells are sensitive, a lower DAR (e.g., DAR 2) may be sufficient and safer. For refractory solid tumors with dense stroma and low antigen density, high-DAR (DAR 8+) constructs are likely to remain the clinical standard. This nuance reflects the current trend to move away from a “one-size-fits-all” approach to a more biologically informed design strategy [29].

## 3. Biological Barriers in Solid Tumors

Biological barriers to ADC efficacy in solid tumors do not arise from a single limitation but from the interplay of tumor-intrinsic heterogeneity, microenvironmental constraints, and transport dynamics, which collectively shape drug distribution and therapeutic response.

### 3.1. Tumor Heterogeneity

In contrast to the relatively uniform target expression observed in hematological malignancies, solid tumors present a far more complex therapeutic landscape characterized by tumor heterogeneity, impaired drug penetration, limited extravasation, and inconsistent antigen expression [54]. Tumor heterogeneity itself is a well-established hallmark of cancer, referring to the presence of distinct subpopulations of cells within a single tumor that differ at genetic, epigenetic, and phenotypic levels [55]. At a mechanistic level, tumor ecosystems are characterized by profound molecular diversity, in which subclonal populations harbor divergent driver mutations and evolve under selective pressures imposed by the tumor microenvironment. These subclones adapt through dynamic pathways, including signaling rewiring, such as NOTCH pathway activation, and the acquisition of secondary genetic alterations, ultimately forming complex and interconnected resistance networks that complicate therapeutic intervention [56]. This heterogeneity is not confined to a single spatial scale but extends from intralesional clonal variation to differences across metastatic sites, reinforcing the challenge of achieving uniform therapeutic targeting [56].

Within the context of antibody-drug conjugates (ADCs), this biological variability directly undermines therapeutic efficacy. ADCs depend on the selective binding of their antibody component to tumor-associated antigens; however, heterogeneous tumors frequently contain subpopulations with low or absent antigen expression. These antigen-negative or antigen-low cells evade ADC targeting, survive initial treatment, and can subsequently repopulate the tumor, thereby contributing to treatment resistance and disease relapse [55]. This heterogeneity is further reshaped by tumor evolution through genetic alterations, epigenetic silencing, phenotypic plasticity such as epithelial-to-mesenchymal transition, and the selective expansion of pre-existing resistant clones under therapeutic pressure [55]. Beyond cellular heterogeneity, microenvironmental factors impose additional physical barriers to ADC delivery. Concurrently, the dense extracellular matrix (ECM), composed of collagen, hyaluronan, and proteoglycans, acts as a structural barrier that restricts ADC penetration and limits effective antibody–antigen interactions. Cancer-associated fibroblasts (CAFs) further exacerbate this effect by remodeling the ECM and promoting stromal fibrosis, thereby creating diffusion barriers that prevent uniform intratumoral distribution of ADCs [55]. Whether the spatial and temporal complexity of tumor heterogeneity can be captured in routine clinical workflows remains an open methodological problem. No validated assay quantifies intratumoral antigen distribution at clinically actionable resolution, and the patterns of heterogeneity that most strongly predict ADC failure have not been formally defined.

### 3.2. Limited Vascular Penetration

This heterogeneity-driven variability in target availability is further compounded by physical barriers that limit ADC delivery within solid tumors. Limited tumor and antigen accessibility constitutes a major barrier to the effective delivery of antibody-drug conjugates (ADCs). Due to the inherently limited ability of antibodies to penetrate solid tumors, only a small fraction of the administered ADC reaches and binds to tumor-specific cells, thereby restricting overall drug delivery and necessitating the use of highly potent cytotoxic payloads to achieve therapeutic efficacy [34,57]. A key mechanistic contributor to this limited penetration is the binding-site barrier introduced in Section 1, whereby high-affinity antibodies are captured by perivascular tumor cells and depleted before they diffuse into the tumor core [34,57].

Because perivascular cells internalize and degrade the conjugate before additional molecules can move beyond the vascular margin, the effective penetration distance is determined by the dose required to saturate these binding sites rather than by systemic exposure [7]. Translating these mechanistic insights into clinical practice has stalled at the measurement step: intratumoral ADC penetration cannot be directly quantified in patients, and dose-optimization strategies that account for tumor-specific delivery constraints are not yet operational.

### 3.3. Interstitial Pressure

Beyond vascular limitations, intratumoral pressure dynamics impose an additional layer of restriction on ADC transport. The tumor microenvironment (TME) further limits the efficacy of antibody-drug conjugates (ADCs) by imposing structural and biophysical barriers to drug delivery. Together, they create a physical microenvironment that limits the uniform intratumoral distribution of ADCs. In particular, the accumulation of hyaluronan (HA) in tumors such as pancreatic and breast carcinomas has been shown to markedly increase interstitial fluid pressure (IFP), thereby altering penetration kinetics and biodistribution patterns. Beyond its structural contribution to interstitial pressure, HA has additionally been described as a ligand for CD44 in tumor-cell signaling contexts beyond ADC delivery, with reported activation of pro-survival pathways and epithelial–mesenchymal transition; these intracellular effects fall outside the biophysical scope of this section [56].

At a mechanistic level, interstitial flow within tumors is governed by gradients in IFP, which are elevated throughout the tumor interior. This elevation arises from multiple factors, including the hyperpermeability of tumor vasculature, which increases fluid extravasation; the dense ECM that impedes fluid movement and drainage; and the collapse of intratumoral lymphatic vessels, which impairs fluid clearance. As a result, IFP within tumors can approach microvascular pressure, effectively eliminating pressure gradients across the vessel wall and within the tumor tissue. This loss of pressure differentials suppresses convective transport, rendering diffusion the primary mechanism of drug delivery for large molecules such as ADCs [58].

This abnormal pressure profile is further complicated by spatial variation at the tumor boundary. While IFP remains elevated within the tumor core, it drops to near-zero levels in surrounding normal tissues, creating a steep pressure gradient at the tumor periphery. This gradient drives the outward flow of interstitial fluid, which can carry therapeutic agents away from the tumor, reducing local drug retention. In addition, this fluid may transport growth factors and tumor cells into adjacent tissues and lymphatic vessels, thereby facilitating metastatic dissemination and further diminishing the effectiveness of ADC-based therapies [58]. The biophysical contribution of IFP to ADC penetration is mechanistically clear, but intratumoral pressure cannot be reliably measured or predicted in patients with current clinical tools, leaving the relationship between IFP heterogeneity and treatment outcomes unresolved at the individual-patient level.

### 3.4. Antigen Heterogeneity and Downregulation

These delivery constraints ultimately converge on antigen-level limitations, where uneven target expression further reduces effective ADC coverage. Antigen heterogeneity represents a major biological barrier to the efficacy of antibody-drug conjugates (ADCs) in solid tumors. Spatial heterogeneity in tumor cells leads to substantial variability in antigen expression across different tumor regions, resulting in uneven target availability for ADC binding and reduced therapeutic coverage [54]. More broadly, intratumoral heterogeneity, driven by clonal evolution, epithelial–mesenchymal transition, lineage plasticity, and treatment-induced selection, generates mixed populations of antigen-positive and antigen-negative tumor cells within the same lesion. This heterogeneity is particularly pronounced in advanced epithelial malignancies and contributes to both primary resistance and relapse following initial ADC response [59]. These observations underscore the importance of spatial tumor biology in ADC development, where binary classification of antigen expression is insufficient to predict therapeutic response. Instead, quantitative and spatial assessment of antigen distribution has emerged as a more relevant determinant of treatment efficacy, particularly in tumors with complex architectural organization [59].

Beyond spatial variability in antigen expression, the limited availability of suitable tumor-specific antigens further constrains ADC design. Ideal targets must be highly expressed on tumor cells while remaining absent or minimally expressed in normal tissues to ensure specificity without toxicity. However, only a restricted pool of such antigens exists, and this limitation is exacerbated by both interpatient and intratumoral heterogeneity. Tumors may downregulate, lose, or modify antigen expression under therapeutic pressure, enabling escape from ADC-mediated targeting and reducing overall treatment efficacy [60]. This challenge is further compounded by dynamic alterations in antigen structure and expression. Mechanisms such as antigen drift, characterized by structural changes in the target antigen, can impair antibody recognition and reduce ADC binding efficiency over time [54]. Antigen downregulation represents a common resistance mechanism, directly reducing antibody binding capacity and impairing ADC activity [55].

Within this context, the bystander effect emerges as a compensatory mechanism that partially mitigates the consequences of heterogeneous target expression. The bystander effect refers to the unintended killing of neighboring antigen-negative cells through the diffusion of cytotoxic payloads released from ADC-targeted cells, resulting in localized rather than systemic toxicity [60,61]. This phenomenon is primarily driven by the sustained activity of highly potent payloads following ADC degradation, allowing cytotoxic agents to diffuse into adjacent cells. While this can enhance therapeutic efficacy in heterogeneous tumors, it also introduces a significant risk of off-target toxicity, particularly when payloads are not rapidly inactivated after release [7,60]. Incomplete ADC processing may further contribute to bystander-related toxicity, as ADCs that are not effectively internalized or degraded can release their payload in unintended locations, thereby contributing to heterogeneous treatment responses [60].

These limitations in antigen accessibility and coverage are further reinforced by the tumor microenvironment (TME), which plays a central role in shaping both antigen accessibility and drug distribution [60]. Functionally, the TME promotes immune evasion, therapeutic resistance, and metastatic dissemination through both biochemical signaling and physical barriers. Immunosuppressive cell populations, inhibitory cytokines, and checkpoint ligand expression impair anti-tumor immunity, while dense extracellular matrix components, hypoxia, and acidic conditions restrict drug penetration and reduce treatment efficacy [60]. When the bystander effect is sufficient to compensate for heterogeneous antigen expression, it remains undefined, and the absence of such a threshold limits both patient selection and the rational design of ADC dosing strategies in heterogeneous tumors. The mechanistic continuity between antigen heterogeneity as a delivery barrier (here) and antigen-level resistance after treatment (Section 5.1) reflects that both phenomena share the same underlying biology, with the distinction lying in temporal context rather than mechanism.

**Figure 2:**
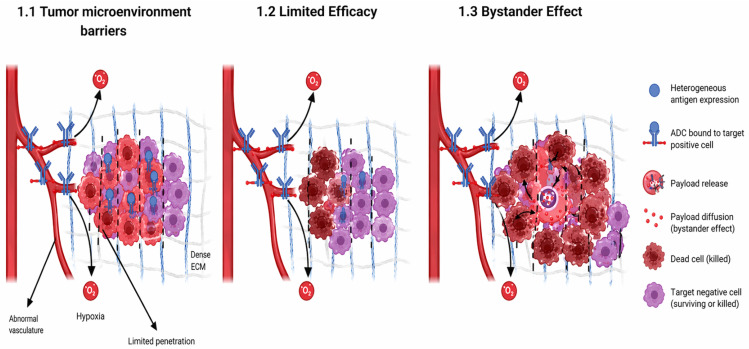
Tumor microenvironment barriers, limited efficacy, and the bystander effect of antibody-drug conjugates (ADCs). This schematic illustrates how the tumor microenvironment can limit the effectiveness of ADCs. Barriers such as abnormal vasculature, dense extracellular matrix (ECM), hypoxia, and heterogeneous antigen expression reduce ADC penetration into the tumor, resulting in uneven drug distribution and lower drug exposure in deeper tumor regions. ADC efficacy may be limited, as only target-positive cells exposed to sufficient ADC concentrations undergo cytotoxicity, while neighboring target-negative or poorly exposed cells survive. The figure also highlights the bystander effect, in which cytotoxic payloads released from target-positive cells diffuse into surrounding cells after ADC internalization and payload release. This diffusion enables the elimination of nearby target-negative tumor cells, partially overcoming the limitations associated with poor penetration and antigen heterogeneity.

Collectively, these barriers do not act in isolation but form an interconnected system in which tumor heterogeneity, vascular limitations, interstitial pressure dynamics, and antigen variability jointly shape ADC delivery and response. A major unresolved challenge is the absence of unified predictive frameworks that integrate these parameters, which continues to limit patient stratification and the rational optimization of ADC-based therapies (Figure 2).

## 4. Clinical Landscape Across Solid Tumors

The approved ADCs for solid tumors and the pivotal trials that established them are summarized in Table 1 and Table 2; the subsections below provide clinical and mechanistic context rather than restating these data.

### 4.1. HER2-Low and Heterogeneous Tumors

HER2-low breast cancer refers to tumors with low but detectable HER2 expression, typically classified as IHC 1+ or 2+ without gene amplification [62]. In this setting, HER2 functions less as a dominant oncogenic driver and more as a docking structure that enables ADC delivery to cells with low receptor density [63]. Trastuzumab deruxtecan (T-DXd) is particularly suited to this context because its membrane-permeable payload and bystander effect allow it to target both HER2-expressing cells and neighboring tumor cells in heterogeneous lesions [63].

Building on this mechanism, the DESTINY-Breast04 trial randomized patients with previously treated metastatic HER2-low breast cancer to T-DXd or the physician’s choice of chemotherapy. The trial enrolled both hormone receptor-positive disease (88.7% of patients) and triple-negative disease (11.3%), with the HR-positive cohort serving as the primary analysis population [64]. T-DXd improved median progression-free survival over chemotherapy in the HR-positive cohort, with a directionally consistent effect in the smaller TNBC cohort [64]. The trial helped define HER2-low disease as a clinically meaningful subgroup rather than simply a subset of HER2-negative tumors [64]. Collectively, the data support a model in which ADC efficacy in HER2-low disease is driven by efficient payload delivery across heterogeneous HER2 expression rather than by high target abundance [63].

However, important limitations remain, as interstitial lung disease, including pneumonitis, is a key toxicity associated with T-DXd and requires vigilant monitoring [64]. Biologically, HER2-low disease remains heterogeneous, and HER2 expression alone may not fully capture underlying tumor behavior or treatment sensitivity [62]. As a result, HER2-low serves as a pragmatic but imperfect biomarker-defined subgroup in clinical practice [62].

### 4.2. Trop-2 Targeting ADCs

Trop-2 is overexpressed in multiple solid tumors and contributes to oncogenic signaling pathways that promote proliferation and metastasis [65]. Sacituzumab govitecan is a Trop-2–directed ADC that links an anti–Trop-2 monoclonal antibody to the topoisomerase I inhibitor SN-38 via a hydrolyzable linker, enabling efficient delivery of the payload [66]. Its extracellular payload release supports a bystander effect in heterogeneous Trop-2 tumors [67].

The phase III ASCENT trial established sacituzumab govitecan as a standard option for metastatic triple-negative breast cancer by demonstrating superior progression-free and overall survival versus physician’s choice chemotherapy [68]. Clinical benefit in ASCENT was observed across multiple subgroups, indicating activity that is not confined to narrowly defined biomarker strata [68]. The TROPiCS-02 trial extended this benefit to heavily pretreated hormone receptor–positive, HER2-negative metastatic breast cancer, confirming the relevance of Trop-2 targeting beyond TNBC [69]. Across ASCENT and TROPiCS-02, sacituzumab govitecan consistently improved progression-free survival compared with single-agent chemotherapy [68]. Overall survival and objective response rates were also higher with sacituzumab govitecan, with the largest absolute progression-free survival benefit observed in the triple-negative cohort of ASCENT [69]. These findings suggest that robust payload delivery and bystander activity can partially overcome the challenges posed by heterogeneous Trop-2 expression [66].

Nevertheless, sacituzumab govitecan is associated with significant toxicity, particularly neutropenia and gastrointestinal adverse events such as nausea and diarrhea [70]. Severe neutropenia and febrile neutropenia are clinically relevant and may require dose modification or treatment interruption [71]. Moreover, Trop-2 expression has shown substantial heterogeneity and inconsistent predictive value, limiting its utility as a reliable biomarker for patient selection [72].

### 4.3. Emerging Targets

HER3 is expressed across breast cancer subtypes and can be exploited for ADC-mediated targeting despite not functioning as a strong independent oncogenic driver in most tumors [73]. Patritumab deruxtecan is a HER3-targeted ADC that uses a cleavable tetrapeptide linker to deliver a topoisomerase I inhibitor payload following receptor-mediated internalization [73]. This design enables the induction of DNA damage and apoptosis in HER3-expressing tumor cells [73].

The phase II single-arm TUXEDO-3 trial evaluated patritumab deruxtecan in 21 patients with metastatic breast cancer and active brain metastases, addressing a setting of high unmet clinical need given the limited intracranial activity of conventional systemic therapy. The trial reported an intracranial objective response rate of 23.8% (5 of 21 patients; 95% CI, 8.2–47.1) according to Response Assessment in Neuro-Oncology Brain Metastases criteria, with responses observed across luminal, HER2-positive, and triple-negative subtypes, supporting subtype-agnostic intracranial activity of HER3-directed ADC therapy [74]. The most common grade 3 or worse treatment-emergent adverse events were neutropenia (14%), diarrhea (10%), and asthenia and vomiting (5% each), with one patient experiencing grade 2 treatment-related pneumonitis [74]. The small sample size and single-arm design limit definitive comparative conclusions, and larger controlled trials are required to define the durability, sequence positioning, and risk-benefit profile of HER3-targeted therapy in this population. A unifying theme across these targets is that modern ADCs increasingly exploit intratumoral heterogeneity rather than being limited by it [62]. HER2-low and Trop-2–directed ADCs show that low or heterogeneous antigen expression can still support effective payload delivery via the bystander effect [63]. HER3-targeted ADCs extend this paradigm into the challenging setting of brain metastases. The evidence base across urothelial, cervical, ovarian, NSCLC, gastric, and colorectal contexts that follows demonstrates that the same linker-payload framework transfers across solid tumor histologies, with clinical efficacy tracking molecular and antigenic context rather than organ of origin [67].

### 4.4. Urothelial Carcinoma and Nectin-4

Nectin-4 is expressed in 60% of bladder tumor specimens with moderate-to-strong staining intensity, with more limited expression in normal epithelial tissue, providing the differential expression that enables ADC targeting [75]. Enfortumab vedotin is composed of an anti–Nectin-4 antibody conjugated to MMAE via a protease-cleavable valine-citrulline linker [75]. The phase III EV-301 trial randomized patients with locally advanced or metastatic urothelial carcinoma whose disease had progressed after platinum-based chemotherapy and a PD-1/PD-L1 inhibitor to enfortumab vedotin or investigator’s choice of chemotherapy [76]. Enfortumab vedotin was the first ADC to demonstrate a significant overall survival advantage in this disease setting [76]. Long-term follow-up of EV-301 confirmed the durability of the survival benefit at 24 months, with no new safety signals [77]. EV-302 then moved the regimen into the first-line setting, randomizing previously untreated patients with locally advanced or metastatic urothelial carcinoma to enfortumab vedotin plus pembrolizumab versus platinum-based chemotherapy [78]. At the primary analysis, the combination significantly improved both progression-free and overall survival, displacing platinum-based regimens from first-line standard of care in this disease [78,79]. The EV-301 to EV-302 progression illustrates the broader pattern this review traces: an ADC moving from later-line salvage therapy into a first-line position once the linker-payload axis proves durable and doing so most effectively in combination with a checkpoint inhibitor rather than as monotherapy [80]. The dermatologic toxicity profile of enfortumab vedotin reflects Nectin-4 expression in normal epidermal keratinocytes and skin appendages [81], illustrated in Section 6, and remains the most clinically relevant management challenge in this regimen.

### 4.5. Cervical and Ovarian Cancer: Tissue-Factor and Folate Receptor Targeting

Two additional ADCs have established meaningful clinical positions in gynecologic malignancies, each exploiting a distinct tumor-associated antigen. Tisotumab vedotin targets tissue factor, an antigen broadly expressed in cervical carcinoma, via an MMAE payload delivered through a protease-cleavable linker. The phase II innovaTV-204 trial demonstrated a confirmed objective response rate of 24% in patients with recurrent or metastatic cervical cancer after platinum-based chemotherapy with or without bevacizumab and a PD-1/PD-L1 inhibitor, establishing the regimen as an active second-line option in a population with limited therapeutic alternatives [82]. The phase III innovaTV-301 trial then randomized similar patients to tisotumab vedotin or the investigators’ choice of chemotherapy and showed a significant overall survival benefit, confirming the ADC’s clinical positioning [83]. Mirvetuximab soravtansine targets folate receptor alpha (FRα), a glycosylphosphatidylinositol-anchored protein highly expressed on the apical surface of high-grade serous ovarian cancer cells. The single-arm phase III SORAYA trial demonstrated an objective response rate of 32.4% in FRα-high platinum-resistant ovarian cancer, supporting accelerated approval [84]. The phase III MIRASOL trial subsequently randomized FRα-positive platinum-resistant patients to mirvetuximab soravtansine or the investigators’ choice of chemotherapy and demonstrated a significant overall survival benefit with the ADC, the first such finding in a heavily treated population where prior agents had produced negligible survival gains [85]. Both agents reinforce the framework developed in Section 2 and Section 3: clinical efficacy follows from the linker-payload axis applied to a target with sufficient differential tumor expression, with the bystander effect of cleavable-linker payloads supporting activity across heterogeneous antigen expression.

### 4.6. Non-Small Cell Lung Cancer

ADC activity in NSCLC has emerged across three distinct molecular contexts, each with a separate biomarker rationale.

In HER2-mutant NSCLC, the phase II DESTINY-Lung01 trial demonstrated a confirmed objective response rate of 55% with trastuzumab deruxtecan in heavily pretreated patients harboring HER2 mutations [86]. The companion HER2-overexpressing cohorts (cohorts 1 and 1A) of the same trial showed lower activity, with a confirmed objective response rate of 26.5% at the 6.4 mg/kg dose, demonstrating that ADC activity in NSCLC is driven by activating HER2 mutations rather than by HER2 protein overexpression alone [87]. DESTINY-Lung02 then compared two dosing levels (5.4 mg/kg and 6.4 mg/kg), establishing the lower dose as the preferred option with a comparable response rate and reduced toxicity [88]. On 11 August 2022, the FDA granted accelerated approval to trastuzumab deruxtecan for HER2-mutant NSCLC based on DESTINY-Lung02 efficacy data, marking the first ADC approval for a HER2-driven solid tumor outside breast and gastric cancer [89]. The approval extended the linker-payload argument across tumor types sharing only a molecular alteration in the same receptor.

In TROP2-expressing NSCLC, the phase III TROPION-Lung01 trial compared datopotamab deruxtecan to docetaxel in previously treated advanced or metastatic disease [90]. Median progression-free survival favored the ADC, with the benefit driven by the non-squamous histology subgroup rather than the squamous subgroup. Median overall survival was numerically higher with datopotamab deruxtecan but did not reach statistical significance in either the overall population or the prespecified non-squamous subgroup, illustrating the importance of histologic context in TROP2-targeted therapy [90].

In c-MET–overexpressing non-squamous NSCLC, the phase II LUMINOSITY trial demonstrated meaningful clinical activity with telisotuzumab vedotin, a c-MET–targeted MMAE conjugate, in patients with previously treated disease [91]. The agent received accelerated FDA approval based on these data, marking the first c-MET–targeted ADC to enter clinical practice [92]. The breadth of ADC activity across HER2-mutant, TROP2-high, and c-MET–overexpressing NSCLC subsets demonstrates that the platform’s clinical utility tracks with biomarker-defined molecular contexts rather than histologic origin, supporting the precision-oncology framing developed in Section 9.

### 4.7. Gastric, Gastroesophageal, and Colorectal Cancer

T-DXd has extended HER2-targeted ADC activity beyond breast cancer into gastric and colorectal malignancies, validating the cross-tumor portability of the linker-payload framework. In HER2-positive advanced gastric or gastroesophageal junction cancer, the phase II DESTINY-Gastric01 trial demonstrated a confirmed objective response rate of 51% with T-DXd versus 14% with physician’s choice chemotherapy in Asian patient’s refractory to trastuzumab-based therapy [93]. The single-arm DESTINY-Gastric02 trial then extended these findings to a Western patient population, demonstrating a comparable response rate (38%) in patients progressing after trastuzumab-based therapy and supporting the agent’s regulatory approval in this setting [94].

In HER2-positive metastatic colorectal cancer, the phase II DESTINY-CRC01 trial demonstrated a 45.3% objective response rate with T-DXd in patients with HER2-positive, refractory disease [95,96]. DESTINY-CRC02 then compared two T-DXd dose levels in HER2-positive metastatic colorectal cancer that had progressed on standard therapy, supporting 5.4 mg/kg as the preferred dose with a 37.8% objective response rate and a tolerable safety profile [97]. These findings establish HER2-targeted ADC activity in a setting where targeted therapy options had previously been minimal and where conventional cytotoxic chemotherapy plateaus rapidly.

### 4.8. Stage Migration: From Refractory Salvage to Earlier-Line Therapy

The clinical trajectory of ADCs has progressively moved earlier in the treatment sequence. The platform first demonstrated clinical value in hematologic malignancies, gemtuzumab ozogamicin in AML and brentuximab vedotin in Hodgkin lymphoma, before extending into refractory metastatic solid tumors with T-DM1 in pretreated HER2-positive breast cancer and T-DXd in DESTINY-Breast03, and is now advancing into earlier-line and curative-intent settings. The KATHERINE trial established T-DM1 as superior to trastuzumab for residual HER2-positive early breast cancer following neoadjuvant therapy, demonstrating that ADC activity translates into improved disease-free survival in the adjuvant setting rather than being confined to advanced disease [98]. DESTINY-Breast05 subsequently demonstrated the superiority of T-DXd over T-DM1 for invasive disease-free survival in residual HER2-positive early breast cancer after neoadjuvant therapy, and DESTINY-Breast11 showed higher rates of pathologic complete response with T-DXd–based neoadjuvant regimens than with standard chemotherapy-based approaches [99,100]. These data position third-generation ADCs as disease-defining therapies in earlier-stage care, where their absolute clinical benefit is likely to be greatest and where the durability of response can translate into long-term cure rather than disease control. The EV-302 advance of enfortumab vedotin into first-line urothelial therapy reflects the same migration pattern in a second solid-tumor context [78].

## 5. Mechanisms of Resistance

Resistance to antibody-drug conjugates (ADCs) arises from coordinated failures across the therapeutic cascade, including target engagement, internalization, intracellular processing, and execution of cytotoxicity. These mechanisms are shaped by heterogeneous drug exposure within tumors, which imposes selective pressures that favor the survival of poorly exposed cell populations. Consequently, resistance emerges as an integrated process involving antigen modulation, trafficking defects, altered drug handling, and evasion of cell death pathways.

### 5.1. Antigen Loss or Heterogeneity

As established in Section 3.1, tumor ecosystems are molecularly heterogeneous, with subclonal driver diversity and signaling rewiring generating resistance networks; during treatment, this heterogeneity evolves and directly shapes ADC efficacy [56]. Within this context, antigen heterogeneity is not static but evolves throughout treatment, directly influencing the effectiveness of antibody–drug conjugates (ADCs).

A central mechanism of ADC resistance involves the downregulation, mutation, or structural modification of target antigens, which impairs antibody binding and reduces intracellular payload delivery. This phenomenon has been consistently observed across multiple ADCs, including trastuzumab emtansine (T-DM1), brentuximab vedotin (BV), and sacituzumab govitecan (SG), where loss or alteration of HER2, CD30, and TROP2 expression, respectively, diminishes therapeutic efficacy. Notably, the TROP2 T256R missense mutation reduces antibody binding affinity in SG-treated triple-negative breast cancer, illustrating how antigen modification directly compromises ADC-target engagement [101].

At a mechanistic level, resistance is further reinforced by alterations in antigen-associated trafficking pathways. Despite apparent antigen expression, functional resistance may arise when internalization or intracellular routing is disrupted. For instance, downregulation of Endophilin A2 (EndoA2) in HER2-positive tumors impairs endocytosis and lysosomal trafficking of HER2-targeted ADCs, limiting intracellular payload release and reducing cytotoxicity [56]. Similarly, alternative endocytic routing has been observed in T-DM1-resistant models, where ADCs are sequestered in caveolin-1-positive compartments, bypassing canonical lysosomal processing and thereby preventing effective payload activation [56]. These findings indicate that the presence of antigen alone is insufficient, as effective ADC activity depends on intact internalization and trafficking pathways. Longitudinal exposure to ADCs further drives antigen loss as an acquired resistance mechanism. Experimental models demonstrate that repeated T-DM1 treatment leads to progressive reduction in HER2 protein expression, while clinical and preclinical studies of BV similarly report decreased CD30 expression in resistant cell lines and patient-derived samples [102]. These observations support a model in which therapeutic pressure selectively enriches antigen-low or antigen-negative subclones, thereby reducing overall tumor susceptibility to ADC targeting.

Paradoxically, high antigen expression may also impair ADC efficacy through systemic antigen-mediated drug sequestration. In the case of gemtuzumab ozogamicin (GO), elevated CD33 expression in peripheral blood acts as a sink that consumes circulating ADC molecules, limiting their bioavailability within the bone marrow and reducing therapeutic exposure at the intended site of action [102]. This highlights a critical limitation in ADC pharmacokinetics, in which both insufficient and excessive antigen expression can adversely affect treatment outcomes. Therapeutic pressure from prior or combination treatments further exacerbates antigen instability. In HER2-positive breast cancer, multiple lines of anti-HER2 therapy have been associated with reduced HER2 expression, leading to diminished T-DM1 binding, impaired internalization, and decreased payload delivery [103]. Similarly, reduced CD30 expression in BV-resistant models correlates with decreased intracellular delivery of monomethyl auristatin E (MMAE), reinforcing the dependency of ADC efficacy on sustained antigen expression [103].

In addition to quantitative changes, qualitative alterations in antigen structure and accessibility also contribute to resistance. Truncated HER2 isoforms such as p95HER2 lack the extracellular domain required for trastuzumab binding while retaining signaling capacity, thereby promoting tumor progression despite ADC therapy. Likewise, MUC4 overexpression can sterically mask HER2 epitopes, preventing antibody binding and reducing ADC efficacy. These mechanisms demonstrate that antigen accessibility, rather than expression alone, is a critical determinant of ADC activity [103]. Collectively, these findings establish antigen loss and heterogeneity as a multi-layered resistance mechanism encompassing quantitative downregulation, structural modification, and trafficking dysfunction. However, a key unresolved challenge is the lack of predictive biomarkers to distinguish tumors that will undergo antigen loss from those that maintain stable expression during therapy, limiting the ability to stratify patients and optimize ADC treatment strategies. These antigen-level alterations propagate downstream by limiting effective internalization and intracellular processing, thereby linking target modulation to subsequent resistance mechanisms.

### 5.2. Impaired Internalization

The therapeutic efficacy of antibody-drug conjugates (ADCs) is fundamentally dependent on efficient internalization and intracellular processing, as the release of the cytotoxic payload requires successful trafficking to lysosomal compartments. In the absence of proper internalization or effective intracellular routing, ADCs fail to achieve sufficient intracellular drug concentrations, thereby limiting their cytotoxic potential [54]. Consequently, alterations in endocytic pathways represent a critical and frequently observed mechanism of resistance. Importantly, under heterogeneous drug exposure imposed by delivery constraints in solid tumors, subpopulations of cells may preferentially survive by adopting alternative trafficking routes that further limit effective payload delivery. Under physiological conditions, most ADCs are internalized via clathrin-mediated endocytosis and subsequently trafficked to lysosomes, where acidic processing facilitates payload release. However, resistant tumor cells can disrupt this canonical pathway, resulting in reduced co-localization of ADCs with lysosomes and diminished cytotoxic efficacy, as observed in T-DM1-resistant models [54]. This shift in intracellular routing highlights that resistance can arise not only from impaired uptake but also from defects in downstream intracellular trafficking.

A key mechanism underlying this phenomenon is the preferential engagement of caveolae-mediated endocytosis. Sung et al. demonstrated that T-DM1-resistant cells, including N87-TM models, internalize trastuzumab-based ADCs via caveolin-1 (CAV1)-associated pathways, resulting in sequestration within CAV1-positive vesicles rather than lysosomal compartments [102]. Given that CAV1 regulates cargo selection and internalization dynamics, its involvement fundamentally alters the intracellular fate of ADCs. [103,104]. Consistently, increased co-localization of T-DM1 with CAV1 has been associated with reduced therapeutic response in HER2-positive cell lines, and similar trafficking-dependent resistance patterns have been observed in other ADC systems, including antimelanotransferrin-based constructs [102]. Notably, the role of CAV1 in ADC resistance extends beyond simple overexpression. While elevated CAV1 levels have been reported in certain resistant cell lines, resistance does not correlate directly with expression levels, as caveolae-mediated uptake has been observed in both sensitive and resistant models following ADC exposure [103,105]. Instead, resistance appears to be driven by the preferential routing of ADCs via caveolae-associated pathways, thereby altering intracellular processing rather than uptake per se. Mechanistically, this is significant because caveosomes maintain a neutral pH, in contrast to the acidic conditions in lysosomes required for efficient payload release, thereby impairing ADC activation and cytotoxicity [103]. However, the precise contribution of caveolae-mediated trafficking to ADC resistance remains unresolved, and further investigation is required to clarify its role in determining therapeutic response.

Beyond pathway selection, disruption of endocytic machinery itself can also impair ADC efficacy. Endophilin A2 (EndoA2), a regulator of clathrin-independent endocytosis, has been shown to facilitate HER2 internalization under physiological conditions. Silencing of EndoA2 inhibits HER2 internalization and reduces the cytotoxic effects of trastuzumab and T-DM1 in HER2-positive cell lines, establishing EndoA2 downregulation as a functional mechanism of resistance [103,106]. These findings underscore the importance of intact internalization machinery in maintaining ADC sensitivity.

In addition to altered uptake pathways, receptor-specific trafficking dynamics further contribute to resistance. Following internalization, receptor-ADC complexes may be directed toward recycling pathways rather than lysosomal degradation. For example, epidermal growth factor receptor (EGFR) complexes can undergo rapid recycling through early endosomes, where the mildly acidic pH is insufficient to release the payload, or follow slower recycling routes via multivesicular body extensions [107]. In contrast, HER2 exhibits limited internalization efficiency and preferentially remains at the plasma membrane following antibody binding, reducing its delivery to lysosomes and thereby limiting ADC processing [103,108]. This intrinsic resistance to internalization represents a fundamental limitation of HER2-targeted ADCs.

Comparative analyses further highlight the impact of receptor trafficking on ADC efficacy. Despite markedly higher surface expression of HER2 relative to CD22, significantly greater intracellular accumulation of the MMAE payload has been observed in CD22-targeted systems, reflecting more efficient lysosomal trafficking of CD22 than HER2 [103]. This disparity suggests that only a small fraction of HER2 is routed to lysosomes, while the majority is recycled back to the cell surface, thereby limiting payload delivery. The mechanisms governing differential receptor sorting between recycling and degradation pathways remain incompletely understood, representing a key unresolved factor in ADC resistance [103,109].

Collectively, these findings establish impaired internalization and altered intracellular trafficking as multifaceted resistance mechanisms encompassing pathway switching, endocytic machinery dysfunction, and receptor-specific routing biases. However, a critical gap remains in defining the determinants governing receptor sorting toward lysosomal versus recycling pathways, thereby limiting the ability to predict ADC responsiveness and to rationally optimize drug selection strategies. As such, impaired internalization functionally converges with antigen loss by preventing effective delivery of the intracellular payload despite preserved surface expression.

### 5.3. Drug Efflux Transporters

A central mechanism underlying resistance to antibody-drug conjugates (ADCs) is the upregulation of ATP-dependent drug efflux transporters, which actively reduce intracellular payload accumulation. This system is primarily mediated by members of the ATP-binding cassette (ABC) transporter superfamily, including P-glycoprotein (P-gp/MDR1), multidrug resistance-associated proteins (MRPs), and breast cancer resistance protein (BCRP/ABCG2), all of which function to export cytotoxic compounds from tumor cells and thereby attenuate ADC efficacy [56]. By lowering intracellular drug concentrations, these transporters directly compromise the cytotoxic activity of ADC-delivered payloads [54]. Under conditions of heterogeneous drug distribution within solid tumors, regions exposed to sublethal payload concentrations may impose selective pressure favoring the survival and expansion of cells with elevated efflux capacity.

Among these, P-glycoprotein remains one of the most extensively characterized mediators of ADC resistance. Overexpression of P-gp is frequently observed in resistant tumor cells and continues to represent a major barrier to the clinical efficacy of ADCs [54,56]. Mechanistically, P-gp recognizes and exports a broad range of hydrophobic cytotoxic payloads, including calicheamicin gamma 1, monomethyl auristatin E (MMAE), DM1, and DM4, all of which are commonly used in ADC platforms [56]. Recent evidence further supports the role of P-gp as a pan-ADC resistance determinant, as P-gp-mediated efflux of the DXd payload significantly reduces the cytotoxicity of datopotamab deruxtecan (Dato-DXd) across multiple cancer cell models [110].

The clinical relevance of P-gp-mediated resistance is supported by observations in hematologic malignancies. In acute myeloid leukemia (AML), cells rendered resistant to gemtuzumab ozogamicin (GO) exhibit increased MDR1 expression, with significantly higher efflux activity observed in non-responders compared to responders. Similar findings have been reported with inotuzumab ozogamicin (IO), which shares the calicheamicin payload, indicating that efflux-mediated resistance can directly influence treatment outcomes [102].

Beyond P-gp, other members of the ABC transporter family contribute to multidrug resistance in ADC-treated tumors. Multidrug resistance-associated proteins (MRPs), particularly MRP1, MRP2, and MRP4, facilitate the ATP-dependent export of xenobiotics and their conjugated metabolites [56]. MRP1, which is widely expressed across multiple cancer types, can export detoxified drug conjugates such as glutathione (GSH)-adducted metabolites, thereby reducing intracellular levels of active drug species and impairing ADC activity [56]. More broadly, MDR1 expression has been associated with poor clinical outcomes in patients treated with calicheamicin-based ADCs, reinforcing its role as a determinant of therapeutic response [101]. Similarly, MRP2 (ABCC2), which is highly expressed in several solid tumors, including pancreatic, colorectal, and gastric cancers, preferentially transports organic anions and drug conjugates. Notably, SN-38, a bioactive metabolite commonly used as an ADC payload, has been identified as an MRP2 substrate, with MRP2-mediated efflux contributing to resistance in pancreatic ductal adenocarcinoma models [56]. Breast cancer resistance protein (BCRP/ABCG2) represents another key efflux transporter implicated in ADC resistance. Structurally related to P-gp and MRP1, BCRP is expressed in multiple tissues, including the liver, intestine, and hematopoietic compartments, and functions as a broad-spectrum drug efflux pump [56]. Recent studies have demonstrated that BCRP actively exports several ADC payloads, including MMAE, SN-38, and DM1, thereby reducing intracellular drug accumulation and diminishing ADC potency [56].

Collectively, these findings indicate that most ADC cytotoxic payloads are substrates of ABC transporters, and that upregulation of these efflux systems represents a major barrier to sustained intracellular drug exposure [103]. Consistent with this, multiple ADC-resistant cell lines, including T-DM1-resistant models, exhibit upregulation of ABC transporter genes, highlighting their role in both cleavable and non-cleavable ADC resistance mechanisms [103]. Moreover, efflux-mediated resistance may be present at baseline or emerge following therapeutic exposure, reflecting both intrinsic and acquired resistance pathways [101]. However, the contribution of efflux transporters to ADC resistance is not uniform across all systems. In certain models, alterations in drug efflux do not fully account for the observed resistance phenotype, suggesting that efflux-independent mechanisms may predominate in specific contexts [103]. This variability underscores the complexity of ADC resistance and indicates that efflux-mediated drug export, while significant, operates within a broader network of resistance pathways.

### 5.4. Resistance to Payload-Induced Cytotoxicity

The cytotoxic efficacy of antibody-drug conjugates (ADCs) ultimately depends on the ability of delivered payloads to induce lethal cellular damage and trigger apoptotic cell death. However, tumor cells can evade apoptosis by altering damage-response pathways and cell-death signaling, even in the presence of effective intracellular payload delivery [54]. Dysfunction of apoptotic signaling is a well-established mechanism of resistance across multiple therapeutic modalities and similarly contributes to reduced ADC efficacy [54]. In this context, inactivating mutations in key tumor suppressor genes such as TP53 and ATM have been identified in cases of resistance to inotuzumab ozogamicin (INO), highlighting the importance of intact DNA damage response pathways in mediating ADC-induced cytotoxicity [54].

At a mechanistic level, resistance arises when tumor cells shift the balance between pro-apoptotic and anti-apoptotic signaling, thereby preventing the execution of cell death programs despite payload-induced damage. Overexpression of anti-apoptotic proteins such as BCL-2 and BCL-XL has been shown to reduce sensitivity to ADCs, including gemtuzumab ozogamicin (GO) and brentuximab vedotin (BV), effectively rendering tumor cells resistant to apoptosis triggered by cytotoxic payloads [54]. These adaptations allow cells to tolerate otherwise lethal levels of intracellular damage, significantly diminishing the therapeutic impact of ADCs [54].

Conversely, impairment of pro-apoptotic signaling further contributes to resistance. The pro-apoptotic proteins BAX and BAK, which are critical mediators of mitochondrial apoptosis, have been implicated in regulating sensitivity to GO in acute myeloid leukemia, with defects in their activation associated with reduced treatment response [102,111]. These findings underscore that both suppression of pro-apoptotic pathways and enhancement of survival signaling converge to disrupt apoptosis and promote resistance. This imbalance in apoptotic regulation is not limited to specific ADCs but represents a broader mechanism of both primary and acquired resistance. Constitutive overexpression of anti-apoptotic proteins such as BCL-2 and BCL-xL, as well as functional deficiencies in BAX and BAK, can impair apoptosis and thereby reduce the cytotoxic effects of ADC-delivered payloads [101]. In non-Hodgkin lymphoma, resistance to polatuzumab vedotin has been associated with decreased expression of the pro-apoptotic protein Bim alongside increased BCL-2 levels, further illustrating how coordinated alterations in apoptotic regulators drive treatment failure [101]. Similarly, elevated BCL-XL expression has been linked to reduced sensitivity to anti-CD79b-valine-citrulline-MMAE, reinforcing the role of anti-apoptotic signaling in mediating resistance across different ADC platforms [102].

Collectively, these findings indicate that even when ADCs successfully deliver cytotoxic payloads, alterations in apoptotic signaling can prevent effective cell death, thereby constituting a critical downstream resistance mechanism. However, a key unresolved challenge is determining which tumors harbor pre-existing defects in the apoptotic machinery versus those that acquire such adaptations under therapeutic pressure, limiting the ability to predict response and to optimize ADC-based treatment strategies.

### 5.5. Lysosomal Dysfunction

The cytotoxic activity of antibody-drug conjugates (ADCs) is critically dependent on successful lysosomal processing, where enzymatic or chemical cleavage of the linker releases the active payload [102]. Disruption of lysosomal function at any stage, including acidification, proteolytic degradation, or catabolite export, can impair payload release and contribute to therapeutic resistance.

A central determinant of lysosomal function is the maintenance of an acidic intraluminal environment. Inadequate acidification, often resulting from impaired activity of the vacuolar H^+^-ATPase (V-ATPase), can significantly reduce lysosomal protease activity, leading to incomplete antibody degradation and impaired payload release [103]. Consistent with this, T-DM1-resistant cells have been shown to exhibit elevated lysosomal pH, which inhibits proteolytic enzyme activity and compromises intracellular ADC processing [54,102]. In controlled experimental models, pharmacological inhibition of V-ATPase with bafilomycin A1—a research tool used to probe lysosomal acidification rather than a translatable therapeutic—was sufficient to induce a T-DM1 resistance phenotype, supporting the dependence of ADC metabolism on intact lysosomal acidification [103]. As V-ATPase activity declines, T-DM1 processing is reduced, ultimately leading to diminished cytotoxic efficacy and the emergence of drug resistance [103].

The impact of lysosomal dysfunction is particularly pronounced for ADCs incorporating non-cleavable linkers, which rely entirely on proteolytic degradation of the antibody backbone to generate active cytotoxic metabolites [103]. In this context, even subtle impairments in lysosomal enzyme activity can markedly reduce payload availability, highlighting the dependence of these constructs on intact lysosomal processing pathways.

Beyond degradation, efficient ADC activity requires the transport of generated catabolites from the lysosomal lumen into the cytosol. The lysosomal membrane protein SLC46A3 plays a critical role in this process by facilitating the export of lysine-MCC-DM1, the active metabolite produced following T-DM1 degradation [54]. Downregulation of SLC46A3 has been consistently associated with reduced efficacy of non-cleavable ADCs, including T-DM1, due to impaired cytosolic delivery of the payload [54,102]. This mechanism has been further validated in multiple models, in which decreased SLC46A3 expression confers resistance to DM1-based ADCs by limiting the intracellular distribution of cytotoxic catabolites, without affecting the activity of certain auristatin-based constructs [103]. These findings underscore that lysosomal export, in addition to degradation, represents a critical determinant of ADC efficacy.

Lysosomal sequestration introduces an additional layer of complexity in intracellular drug handling. Weakly basic and hydrophobic cytotoxic agents can diffuse from the cytoplasm into lysosomes, where they become protonated and are trapped in the acidic environment. This process leads to intracellular drug accumulation in lysosomes rather than its distribution to cytosolic targets, thereby reducing pharmacological activity [103]. Modulation of lysosomal pH through V-ATPase inhibition can reverse this sequestration, allowing the redistribution of trapped compounds into the cytoplasm and restoring drug activity [103]. These observations highlight the dual role of lysosomal acidification in both enabling payload release and influencing intracellular drug distribution.

Importantly, the dependence of ADC processing on specific lysosomal enzymes varies according to linker design. For ADCs incorporating valine–citrulline linkers, the generation of active catabolites can occur through multiple enzymatic pathways, and cathepsin B is not strictly required for their processing [102]. This suggests that resistance arising from lysosomal dysfunction may differ across ADC classes, depending on their reliance on specific enzymatic or chemical cleavage mechanisms. Collectively, lysosomal dysfunction represents a multi-dimensional resistance mechanism encompassing impaired acidification, reduced proteolytic activity, defective catabolite export, and altered intracellular drug sequestration. However, a major unresolved challenge remains the limited understanding of how lysosomal transport and processing pathways are selectively altered in resistant tumor cells, restricting the ability to predict ADC response based on lysosomal function.

**Figure 3:**
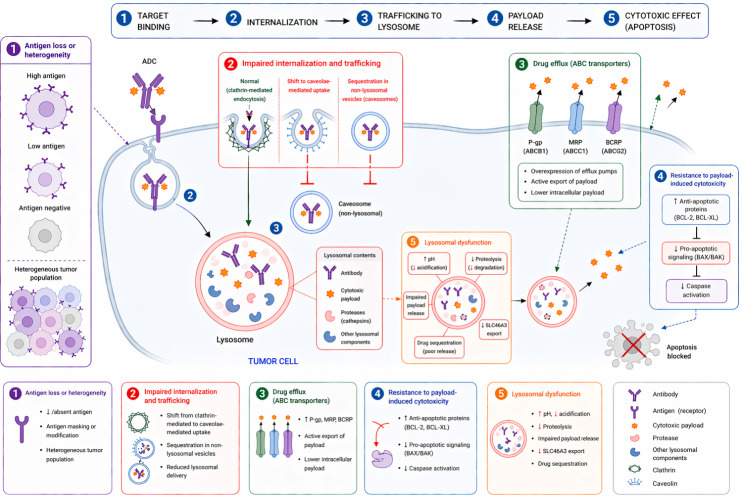
Mechanisms of resistance to antibody-drug conjugates (ADCs). This schematic illustrates the ADC therapeutic cascade, from target binding and internalization to lysosomal processing, payload release, and induction of apoptosis, and highlights key resistance mechanisms that disrupt this pathway. Antigen loss or heterogeneity limits ADC binding through reduced, absent, or structurally altered target expression. Impaired internalization and trafficking redirect ADCs away from lysosomes via caveolae-mediated uptake and sequestration in non-degradative compartments, reducing effective payload delivery. Lysosomal dysfunction, including impaired acidification, reduced proteolysis, defective SLC46A3-mediated export, and drug sequestration, further compromises payload release. Drug efflux transporters (P-gp, MRP, BCRP) actively export cytotoxic payloads, lowering intracellular drug accumulation. Finally, resistance to payload-induced cytotoxicity arises from dysregulated apoptotic signaling, characterized by increased anti-apoptotic proteins (BCL-2, BCL-XL), reduced pro-apoptotic activity (BAX/BAK), and impaired caspase activation.

Taken together, resistance to antibody-drug conjugates reflects a coordinated failure across the ADC lifecycle rather than a single dominant mechanism. Antigen modulation limits target engagement, defects in internalization and trafficking restrict lysosomal delivery, efflux transporters reduce intracellular drug accumulation, and alterations in apoptotic signaling prevent effective cell death. These mechanisms are functionally interconnected, such that disruption at one stage reinforces resistance at others, ultimately converging on insufficient intracellular drug exposure and diminished cytotoxicity. This integrated model underscores the combined influence of heterogeneous drug delivery and tumor-intrinsic adaptations, while highlighting the absence of predictive frameworks capable of resolving these interacting resistance layers in individual tumors (Figure 3).

## 6. Toxicity and Safety Profiles

### 6.1. On-Target/Off-Tumor Toxicity

Although several antibody-drug conjugates have achieved regulatory approval, their clinical use remains limited by meaningful toxicity [112]. On-target/off-tumor toxicity occurs when the antigen is present not only on malignant cells but also at low levels in normal tissues, allowing the ADC to bind healthy cells and deliver its payload there. This mechanism can produce tissue-specific adverse effects depending on where the antigen is physiologically expressed [112].

Clinical observations suggest that this toxicity mechanism contributes to certain organ-specific adverse events when target antigens are also present in normal tissue [113]. For example, Nectin-4 expression in normal skin has been linked to dermatologic toxicity in patients treated with enfortumab vedotin. This shows that target distribution in healthy tissue can shape the toxicity profile of an ADC [113]. Overall, on-target/off-tumor effects are less common than payload-related toxicity, but they remain important because they explain specific adverse events tied to antigen expression in normal tissue. This mechanism should therefore be considered in target selection, ADC design, and clinical monitoring [112].

### 6.2. Payload-Driven Toxicity

A large share of ADC toxicity is driven by the cytotoxic payload rather than the target antigen itself [112]. Dose-limiting toxicities often recur across ADCs that share the same payload, even when they target different antigens or tumor types, highlighting the payload as a primary determinant of toxicity profiles. Across approved ADCs, rates of dose reduction, treatment delay, discontinuation, and grade 3 or higher adverse events are often similar across agents with the same payload class [113]. Hematologic and gastrointestinal toxicities are especially common and tend to reflect the payload mechanism rather than antigen expression. This pattern supports the idea that payload potency and systemic exposure are central to ADC tolerability. For example, grade 3 or higher diarrhea has been reported in approximately 10% of patients treated with sacituzumab govitecan, consistent with its SN-38 payload derived from irinotecan [113]. Together, these observations show that payload properties are key determinants of the therapeutic window.

### 6.3. Hematologic Toxicity

Hematologic toxicity is one of the most common adverse effects of ADC therapy, frequently appearing as neutropenia, anemia, or thrombocytopenia [114]. These toxicities are seen across multiple ADCs and represent a major component of their safety burden. The pattern of hematologic toxicity varies by agent. Thrombocytopenia is more prominent with trastuzumab emtansine, anemia is more frequent with trastuzumab deruxtecan, and neutropenia is a key toxicity with sacituzumab govitecan [114]. This suggests that different ADCs produce distinct hematologic toxicity profiles even when these events are broadly shared. Severe hematologic toxicity can also limit treatment delivery. In a study of MEDI7247, thrombocytopenia, neutropenia, and anemia were the most frequent adverse events and the most common grade 3 or 4 toxicities [115]. These toxicities were dose-limiting and restricted repeated administration of the drug.

Overall, hematologic toxicity is central to ADC tolerability and often necessitates dose modification or interruption, remaining a main practical constraint on long-term ADC use [115].

### 6.4. Strategies to Mitigate Toxicity

Toxicity mitigation for ADCs relies on dose adjustment, supportive care, and close monitoring [116]. Approved products include drug-specific guidance for dose reduction, interruption, or discontinuation when adverse events occur. These measures are designed to preserve benefit while limiting treatment-related harm. Supportive care is also important because several toxicities are reversible when recognized early. Multidisciplinary care can improve management by enabling early specialist input, such as ophthalmologic assessment when ocular toxicity occurs [116]. This underscores proactive monitoring as a central component of safe ADC administration. Some toxicities require specific management. Interstitial lung disease and pneumonitis associated with trastuzumab deruxtecan require prompt treatment interruption or discontinuation and may also require corticosteroid therapy [117]. Because these events can become severe or fatal, early recognition is essential. Overall, toxicity mitigation depends on early detection, individualized dosing, and coordinated supportive care, all of which are critical for maintaining the balance between efficacy and tolerability in ADC therapy [117].

## 7. Next-Generation ADC Strategies

### 7.1. Bispecific ADCs

Bispecific ADC (bsADC) design emerged to address antigen escape. By targeting two distinct antigens on the same tumor cell (e.g., HER2 and HER3), this ADC configuration has been shown to prevent tumors from developing resistance by downregulating a single receptor [33,118]. This “dual-targeting” approach also enhances the specificity of the drug; bsADCs can be engineered to release their payload only when both targets are engaged, which could significantly reduce toxicity in healthy tissues that express only one of the antigens [119,120]. Mechanistically, bsADCs can trigger synergistic internalization. In some cases, binding to two different receptors causes them to cluster and endocytose much faster than binding to a single receptor would [33,120]. This rapid internalization is a major clinical advantage, as it overcomes the limitations of receptors with slow turnover rates or that typically recycle to the surface [118]. Another trend in bsADCs is the “biparatopic” format, which targets two different epitopes on the same antigen. This has been shown to induce massive receptor cross-linking and degradation, effectively shutting down the signaling pathways that the tumor uses for growth while simultaneously delivering the cytotoxic payload [29,33]. This is a promising strategy for patients who have progressed on standard monospecific ADCs [33,120].

### 7.2. Immune-Stimulating ADCs

Immune-stimulating Antibody Conjugates (ISACs) represent the “next frontier” by shifting the paradigm from direct cell-killing to immunological re-education. Instead of a toxin, these ADCs deliver innate immune agonists directly to the tumor microenvironment, with two mechanistically distinct payload classes. Toll-like receptor (TLR) agonists engage endosomal pattern-recognition receptors and signal through MyD88- or TRIF-dependent pathways to drive NF-κB and type I interferon responses, whereas STING agonists activate the cytosolic cGAS-STING axis through direct binding to the STING adaptor and signal primarily through TBK1-IRF3 to induce type I interferon [49]. This triggers an in situ vaccine effect, in which the tumor’s own antigens are presented to the immune system, leading to a systemic T-cell response [33,121]. For clinicians, the primary appeal of ISACs is the abscopal effect—the ability to treat a local tumor and see regression in distant, non-targeted metastases [49]. This is especially valuable for “cold” tumors that are traditionally resistant to checkpoint inhibitors. By activating local innate immune cells (such as dendritic cells and macrophages), ISACs generate the necessary inflammatory signals to recruit T cells into the tumor [120,122].

### 7.3. Combination Therapies

The synergy between ADCs and Immune Checkpoint Inhibitors (ICIs) is perhaps the most active area of clinical research. ADCs induce immunogenic cell death (ICD), releasing damage-associated molecular patterns (DAMPs) and neoantigens [123]. These priming exposes tumor-associated antigens to antigen-presenting cells and enhance T-cell recognition, increasing susceptibility to PD-1 or CTLA-4 checkpoint blockade [29,124]. This combination is already showing remarkable efficacy in urothelial and breast cancers [28,124]. ADC + Targeted Therapy is another emerging direction. Combining an ADC with a tyrosine kinase inhibitor (TKI) can block bypass signaling pathways that the tumor might use to survive the ADC’s payload [125]. Furthermore, some TKIs can increase the expression of the ADC’s target antigen, increasing the surface antigen pool available for ADC binding [33,37]. This combination engages tumor cells through both surface antigen targeting and inhibition of intracellular signaling [29,125]. ADC combination with pathway inhibitors follows a similar rationale, with the PI3K/AKT pathway providing the strongest biological case. The pathway is a central regulator of proliferation, survival, and angiogenesis in breast cancer, and aberrant activation through PIK3CA mutations or upstream receptor signaling contributes to resistance against endocrine and HER2-directed therapies [126,127]. The phase III CAPItello-291 and SOLAR-1 trials established the clinical activity of PI3K/AKT inhibition in molecularly selected populations through capivasertib-fulvestrant and alpelisib-fulvestrant combinations, respectively [128,129]. These trials do not include ADC arms, but they define a rationale for future ADC combination development in HER2-low or HR-positive disease, where pathway-mediated resistance to ADC therapy is a recurring obstacle and the cytotoxic payload could complement, rather than overlap with, the cytostatic effect of pathway blockade. Toxicity remains a limiting factor: diarrhea, hyperglycemia, and rash with alpelisib, and similar metabolic complications with capivasertib, can necessitate dose reduction; clinical benefit depends on specific molecular alterations, restricting application to genomically defined subgroups [128,129].

### 7.4. Site-Specific Conjugation Technologies

The move toward site-specific conjugation is the technical foundation of “Third Generation” ADCs. Technologies such as THIOMAB, enzymatic GlycoConnect, and unnatural amino acid (uAA) incorporation enable placement of the payload in the most stable and effective location on the antibody [52,53]. This eliminates the “stochastic” nature of earlier drugs, resulting in a homogeneous drug product that behaves more consistently in patients [38,43]. Site-specific conjugation also allows placement of payloads within “protected” pockets of the antibody. This shields the linker from circulating proteases, reducing the risk of premature payload release in the bloodstream [52,130]. This technical advancement is directly responsible for the reduction in systemic toxicities, such as neutropenia and thrombocytopenia, observed in newer ADC trials [33,38].

### 7.5. Non-Internalizing ADCs

The paradigm of non-internalizing ADCs challenges the long-held belief that an ADC must enter the cell to work. These drugs target components of the tumor stroma or the extracellular matrix (ECM), such as fibronectin or collagen [131,132]. Once bound to the stroma, the payload is released in the extracellular space via specialized triggers. Because the payload is lipophilic, it then diffuses into the surrounding tumor cells [131]. This strategy is a powerful tool for overcoming the “antigen barrier” in large solid tumors. Since the ADC does not need to navigate the dense, high-pressure interior of a tumor to be endocytosed, it can deliver its payload to the “periphery,” which then acts as a drug reservoir for the entire tumor mass [29,132]. This approach is currently being explored for “difficult-to-treat” cancers with poor vascularization and low receptor expression [33].

## 8. Translational Challenges and Opportunities

Despite the rapid clinical growth of ADCs, major translational barriers still limit their consistent implementation in routine practice [133]. The primary translational challenge has shifted from the lack of candidate biomarkers to the discordance between biomarker theory and clinical practice [133]. The development of ADCs has progressed faster than the development of biomarkers [133]. This means that patient selection still relies heavily on immunohistochemistry, a semi-quantitative tool prone to threshold effects, sampling bias, and inconsistent correlation with functional drug delivery [134,135,136]. These limitations are most significant in low-expression conditions like HER2-low and HER2-ultralow disease, where slight variations in scoring can affect treatment eligibility, and in metastatic contexts, where biomarker status may change among lesions or over time [135,136,137,138]. The central limitation in patient selection is not the absence of a biological rationale, but the instability of the methods used to measure, interpret, and apply target expression data in clinical practice [134,135,136,138].

Mapping resistance biomarker profiles across patient populations reveals an identical dilemma. Although tissue biopsy provides valuable biological insights, it does not lend itself well to serial assessments in metastatic settings, whereas plasma biomarkers have proven less problematic but have yet to achieve sufficient standardization to compensate for the deficiencies of tissue-guided management [136,139,140,141]. Further complexity arises from the open question of drug sequencing [142]. As ADC drugs become more widely used, there exists uncertainty about how prior ADC exposure affects subsequent responses, especially when the drugs belong to the same class or utilize shared pathways [142,143]. Thus, ADC therapy is evolving faster pharmacologically than the field is developing the tools required to track, sequence, and adapt to its changes [133,142,143]. The challenge posed by manufacturing complexity is another translational obstacle that is frequently overlooked [144,145]. ADC technology is not merely a matter of producing a monoclonal antibody with an extra payload but rather of managing multiple aspects of the process, including the unconjugated antibody, the linker and payload chemistry, and the conjugate itself [23,146,147]. Regulatory guidelines now recognize this and have highlighted the importance of considerations relating to bioanalysis, dosing, pharmacokinetics, immunogenicity, corrected QT interval, and drug–drug interactions [147]. In short, while translational science may involve finding the correct target, the manufacturing complexity of a structurally heterogeneous product must be carefully managed, since efficacy and toxicity cannot be determined from the antibody alone [146,147].

Cost and access represent the last barrier separating scientific advance from real-world benefit [148]. The case of HER2-low trastuzumab deruxtecan serves as an example here: following the identification of a new patient cohort from within an existing one based on a novel biomarker, not only has uncertainty been added to the areas of pathology, but also to those of treatment regimens, payment systems, and health economic assessments [148,149]. Even if a drug demonstrates efficacy, adoption could be restricted either because cost-effectiveness is unclear or because the necessary processes cannot keep up with the science [148]. More generally, it should be recognized that the translation of scientific advances into benefits does not stop with the design of better ADCs [133,147,148].

## 9. ADCs and Precision Oncology

Antibody-drug conjugates (ADCs) have pushed precision oncology beyond a simple “target present or absent” model toward a more dynamic biomarker framework [1]. Increasingly, therapeutic selection depends not only on whether a target is expressed, but also on its abundance, spatial distribution, and disease context [141,150]. This shift is already reflected in regulatory practice: trastuzumab deruxtecan-based strategies are now tied to specific HER2-defined settings, datopotamab deruxtecan is labeled for hormone receptor-positive/HER2-negative disease, and telisotuzumab vedotin requires high c-Met overexpression [92,137,151]. In that sense, biomarker thresholds are no longer peripheral descriptors but are becoming part of the ADC’s therapeutic identity [1].

The HER2-low paradigm shift is the clearest example of this transition [64]. HER2-low should not be viewed simply as a new clinical subgroup, but as evidence that the traditional HER2-positive versus HER2-negative dichotomy was biologically too rigid [64,136]. DESTINY-Breast04 established that trastuzumab deruxtecan could improve outcomes in tumors historically managed as HER2-negative, while DAISY showed that HER2 assignment is not a fixed label but a biologically fluid variable that may shift with sampling and reassessment [64,136]. Subsequent expansion of trastuzumab deruxtecan into HER2-ultralow disease reinforced the same point: as ADC biology has advanced, rigid pathological categories have become less reliable for identifying who may benefit [137,152].

This shift has major implications for biomarker evaluation. If HER2-low exposed the limits of the old classifier, spatial heterogeneity exposed the limits of the biopsy itself [150]. In metastatic disease, a single tissue sample may not adequately represent lesion-level target distribution across the full disease burden [150,153]. In ZEPHIR, whole-body HER2 imaging identified discordance between tissue-defined HER2 positivity and actual lesion-level target expression, helping predict which patients were unlikely to benefit from T-DM1 [150]. Supporting feasibility data with ^89^Zr-trastuzumab PET further suggest that molecular imaging can be clinically useful when standard HER2 assessment is inconclusive [153]. Imaging, therefore, should be viewed not as confirmatory decoration, but as a functional tool for mapping target heterogeneity at the whole-body level [150,153].

Liquid biopsy extends the same precision-oncology logic into the temporal domain [141]. Imaging helps define the distribution of target expression, whereas circulating biomarkers may help track how tumor biology evolves during treatment [141,150]. In DESTINY-CRC01, higher baseline HER2-related markers in tissue and blood were associated with trastuzumab deruxtecan activity, and on-treatment circulating tumor DNA dynamics correlated with response and progression-free survival [141]. However, these findings remain exploratory and are not sufficient to replace tissue-based classification in routine practice [141]. At present, the main value of liquid biopsy lies not in supplanting pathology, but in supporting a more longitudinal approach to ADC selection and resistance monitoring [141]. Overall, ADC precision oncology is moving away from static target identification and toward dynamic target management, where quantitative thresholds, spatial heterogeneity, and temporal evolution all inform therapeutic decision-making [1,136,141,150].

## 10. Precision Immunosuppression: Opportunities and Barriers in Autoimmune Disease

The clinical maturation of ADCs in solid and hematologic cancers has established a template for selective cytotoxic delivery. Whether that template generalizes to non-destructive therapeutic goals remains open. This section examines the rationale for this paradigm, the target and payload choices it demands, and the translational obstacles that have kept it short of routine practice.

### 10.1. Rationale

The case for moving from cytotoxic destruction to selective modulatory paradigms begins with the problem of glucocorticoids. GCs have been central to rheumatoid arthritis management since the 1940s, yet how they should be prescribed remains unresolved; their toxicity is debated, and the field has not settled into consensus on their role [154]. Both European and American guidelines respond by limiting prescribed exposure. European recommendations permit short-term use at treatment initiation or as bridging when a csDMARD is changed, discourage continuation beyond three months, and allow six months only in special cases [155]. The 2015 ACR position is parallel: GCs accompany DMARDs during disease flares, with instruction to minimize dose and duration [154]. Neither body offers granular advice on dose regimen, duration, or administration route because the supporting evidence remains scarce [154]. The toxicity those recommendations aim to contain was cataloged by the 2007 EULAR task force, which identified the principal adverse event categories as cardiovascular, infectious, gastrointestinal, psychological, endocrine, dermatologic, musculoskeletal (osteoporosis among them), and ophthalmologic [156]. Pooled trial data from 2009 estimated adverse event rates above 40 per 100 patient-years even at doses under 30 mg/day [157].

ADCs approach the same problem from the opposite direction. Their oncology track record rests on antibody-directed restriction of payload exposure: a monoclonal antibody recognizes a chosen surface antigen, and the cytotoxic payload reaches only the cells expressing it [54]. That delivery logic transfers to autoimmunity because the relevant surface antigens overlap. CD19 is expressed on the B-cell populations that drive B-cell lymphoma and acute lymphoblastic leukemia, and on the aberrant B cells of systemic lupus erythematosus and rheumatoid arthritis; CAR-T therapy directed at CD19, developed first in the hematologic setting, has since been applied in autoimmune disease [158,159]. Beyond CD19, the convergence is mechanistic: cancer and autoimmune disease share overlapping immunological pathways, and ADC development has accordingly extended beyond oncology, with early clinical evidence now accumulating in autoimmune indications [160,161]. What must change in this transfer is not the delivery architecture but the payload. ADC development is moving beyond the traditional targeted-killing paradigm [54]. Which antigens these platforms should target, and why, is the first question that follows.

### 10.2. Target Landscape in Autoimmunity

The lineage-specific logic starts with the biology. B-lineage cells drive the inflammatory process in rheumatoid arthritis, and depleting them with the chimeric anti-CD20 monoclonal antibody rituximab yields clinical benefit [162]. The pathogenic role of these cells is not confined to the circulation. Autoreactive B cells and plasma cells in RA occupy niches within the inflamed synovium and the bone marrow, where long-lived plasma cell survival is sustained, and the synovium of established RA carries CD20-positive and CD38-positive fractions with CD38 expression notably elevated relative to healthy controls [163]. Rituximab in RA patients with inadequate methotrexate response, given as two infusions alone or combined with cyclophosphamide or continued methotrexate, produced significant symptomatic improvement at weeks 24 and 48 [164].

CD20 itself is expressed on most B-lineage cells but not on pro-B cells or plasma cells, and anti-CD20 depletion splits into two classes: type I agents, including rituximab, and type II agents, including obinutuzumab [163]. The two classes produce different depletion patterns with different clinical consequences. In RA lymph nodes, rituximab clears naïve, unswitched memory, and follicular B cells but leaves switched memory B cells intact, a residue associated with relapse [163]. Obinutuzumab produces more complete and longer-lasting B-cell depletion than type I agents in SLE and RA samples, mediated through both enhanced ADCC, conferred by Fc afucosylation, and a direct, non-apoptotic homotypic adhesion–dependent cell death characteristic of type II anti-CD20 antibodies [165]. In the NOBILITY RCT, it delivered a superior complete renal response over placebo when added to standard therapy, accompanied by reductions in serum IgM and anti-dsDNA, normalization of complement, and stable IgG consistent with survival of CD20-negative plasma cells despite durable depletion of naïve and memory B cells and plasmablasts; a phase III trial is ongoing [163]. The CD20 lineage extends into MS as well, where rituximab has been used off-label and several type I agents have been evaluated: ocrelizumab, with 2–5 fold greater ADCC than rituximab, reduced relapse rate, MRI progression, and markers of neuronal damage across OPERA I/II and ORATORIO and has been approved; ofatumumab, binding a distinct epitope with greater CDC activity, suppressed relapses and progression in ASCLEPIOS I/II; and ublituximab, mediating depletion largely through ADCC, reduced relapses, progression, and radiologic activity over 48 weeks of a phase II study [163].

CD19 is the other lineage-defining target. It is expressed from early bone marrow B-cell development through the plasma cell stage, though a subset of plasma cells is CD19-negative, and it functions within the B-cell receptor signaling complex by aggregating engaged BCRs into surface microclusters [163]. Its regulation is tight in both directions: upregulation can break peripheral tolerance and drive autoimmunity, while downregulation impairs memory B-cell and antibody-titer maintenance [163]. That expression profile makes CD19 a candidate for pan-B-cell depletion, and inebilizumab, an afucosylated anti-CD19 monoclonal with strong ADCC, was safe in a phase I MS trial and reduced lesion formation, though this evidence base remains limited to early-phase MS data [163]. The broader conclusion from this therapeutic space is that recent B-lineage-directed strategies have improved clinical manifestations with limited safety issues [163].

A second class of targets operates through pathways and cell populations active in ongoing inflammation rather than entire cell lineages. OX40 is one such target. A T-cell co-stimulatory molecule in the TNF receptor superfamily (TNFRSF4), OX40 and its ligand OX40L are upregulated in atopic dermatitis, and because the pathway governs expansion, differentiation, and survival of effector and memory T cells, targeting it offers a route to sustained suppression of pathogenic T cells and the inflammation they drive [166]. Antibodies against OX40 (rocatinlimab and telazorlimab) and against OX40L (amlitelimab) have produced early-phase trial data in moderate-to-severe atopic dermatitis [166]. The mechanistic reach of OX40 targeting goes beyond a single cytokine axis: the pathway supports Th1, Th2, Th17, and Th22 subsets and their memory counterparts, so blocking it can reduce pathogenic T cells of multiple lineages and achieve broader disease control than inhibition of any single cytokine they produce [166].

CD64 applies the same pathway-driven logic to the myeloid compartment. A newer class of CD64-directed immunotherapeutics is being developed for macrophage-driven chronic inflammatory disease, including chronic cutaneous inflammation, rheumatoid arthritis, and chronic diabetic wounds [167]. The selectivity these agents achieve is functional rather than binary. Both M1 pro-inflammatory and M2 anti-inflammatory macrophages express CD64, with M2 expressing the receptor at low levels, yet the agents preferentially eliminate the M1 population [168]. This selectivity is thought to stem from greater proteolytic degradation of the constructs within M2 macrophages [167]. Beyond elimination, CD64-directed delivery also opens a modulatory avenue: immunomodulatory proteins delivered to M1 macrophages via CD64 could shift or repolarize the M1/M2 balance and support the resolution of chronic inflammation, a mechanistic alternative to long-used anti-inflammatory drugs such as glucocorticoids, whose efficacy has come with systemic off-target effects [167]. Preclinical hurdles in the development of recombinant immunotoxins remain unresolved [167]. Target selection answers only the first design question. The second is what gets delivered once the antigen is engaged, and which payload classes the field has advanced into human testing.

### 10.3. Engineering Modulatory Payloads

Glucocorticoid-ADCs combine the immunosuppressive potency of glucocorticoids with the precision of biologic delivery. Conventional systemic GCs activate glucocorticoid receptors broadly across tissues; the GC-ADC design routes the steroid payload to immune cells instead, producing localized immunosuppression while reducing the metabolic, skeletal, and adrenal toxicities associated with chronic systemic exposure [169]. The following two trials define what this payload class has achieved in RA. ABBV-3373, composed of adalimumab linked to a glucocorticoid receptor modulator, was evaluated against adalimumab in a Phase IIa proof-of-concept trial in patients with moderate-to-severe RA and inadequate methotrexate response [170]. At week 12, the ADC produced a DAS28-CRP reduction of −2.65 against a historical adalimumab comparator of −2.13 (*p* = 0.022), and 70.6% of ABBV-3373 responders who reached DAS28-CRP ≤ 3.2 at week 12 maintained that response at week 24 despite switching to placebo [170]. Four serious adverse events occurred in the ABBV-3373 arm against two in the adalimumab arm [170]. The investigators concluded that the data supported continued development of the TNF-GRM ADC platform with potential for superior outcomes over existing therapies [170].

ABBV-154 is the successor program in the AbbVie ADC pipeline, similarly composed of adalimumab linked to a glucocorticoid receptor modulator but with engineering changes intended to improve the linker-payload performance relative to ABBV-3373 [171]. It was advanced into a Phase 2b randomized placebo-controlled trial in 472 patients with moderately-to-severely active RA and inadequate response to biologic or targeted synthetic DMARDs. All four ABBV-154 dose groups produced ACR50 response rates above placebo at week 12: 25.5% at 40 mg Q2W, 33.3% at 150 mg Q2W, 44.4% at 340 mg Q2W, and 30.9% at 340 mg Q4W, against 6.3% on placebo (*p* < 0.001 for all comparisons) [171]. Secondary endpoints across ACR, DAS28-CRP, CDAI, and HAQ-DI scores also favored ABBV-154 over placebo across most dose groups [171]. Despite these outcomes, the sponsor terminated the study early and discontinued clinical development of ABBV-154 on the grounds that response rates fell below the hypothesized efficacy threshold and the benefit-risk profile did not differentiate the ADC from existing RA therapies; the decision was not safety-driven [171]. The results were nevertheless framed as informative for future ADC design with alternative linkers or GRM payloads in inflammatory disease [171]. The ADC-GC payload class has therefore cleared the hurdle of placebo-controlled efficacy in autoimmune disease but has not cleared the higher hurdle of clinically meaningful differentiation from existing therapies. That hurdle exists because autoimmune translation operates under constraints that the oncology ADC field has never faced.

### 10.4. Translational Challenges

In chronic rheumatic disease, the comparator is not death but a managed condition, the exposure is not months but years, and the bar for a new agent is not efficacy against placebo but meaningful differentiation from an already large set of working options. Refractory RA is the field’s central unmet need, yet only 10–15% of patients meet that definition, and fewer than half reach remission on existing therapies [172]. This is the evidentiary terrain that ABBV-154 entered and did not leave: the ADC had yet to meet placebo-controlled efficacy endpoints, and the sponsor discontinued development on the grounds that the benefit-risk profile did not differentiate it from available therapies [171]. The safety-efficacy paradox in autoimmune ADC development is therefore calibrated against a crowded shelf, not an empty one. Immunogenicity compounds this calibration. In the same 12-week placebo-controlled window, 32.1% of evaluable ABBV-154 patients developed treatment-emergent anti-drug antibodies and 7.8% developed neutralizing antibodies [171]. Autoimmune dosing operates over years, and the 12-week window does not resolve what anti-drug antibody formation looks like across that horizon.

Infection risk follows a similar logic. Ten serious infections occurred across the ABBV-154 arms, compared with none on placebo, during the placebo-controlled period, a pattern the investigators attributed in part to glucocorticoid effects and in part to the pandemic context in which the trial was conducted [171]. The broader biologic literature in RA frames this signal. Pooled evidence from 42,330 patients across 106 RCTs places the absolute increase in serious infections at approximately 6 per 1000 for standard-dose biologic therapy, 17 per 1000 for high-dose biologic therapy, and 55 per 1000 for combination biologic regimens, against a baseline DMARD rate of roughly 20 per 1000 per year [173]. Non-randomized data suggest the risk is front-loaded, concentrated early in treatment rather than accumulating linearly [173]. For an ADC platform proposing lifetime dosing in autoimmune indications, this matters. Short trials may capture a peak rather than a floor. The section opened by asking whether the oncology ADC template generalizes to non-destructive therapeutic goals. The evidence is incomplete. ADC-mediated immunomodulation can produce placebo-controlled efficacy in autoimmune disease. Whether it can clear the differentiation bar that existing therapies have set, characterize the immunogenicity that emerges within weeks of dosing over the years a chronic indication requires, and manage the infection burden that biologic immunosuppression carries remains an open question for the field as of 2026.

## 11. Future Directions

Whereas Section 7 catalogs the engineering and combinatorial strategies that define the current generation of ADCs, this section examines the platform’s near-term evolution beyond those current-generation tools. These trajectories shift the design problem from delivering cytotoxic payloads with improved tolerability to integrating multiple therapeutic functions within a single molecular construct [174,175]. The first trajectory broadens the ADC concept from selective cytotoxicity to selective immunomodulation, with the immune-stimulating constructs discussed in Section 7.2 as an early instantiation. Preclinical and early clinical work with tumor-targeted STING agonist ADCs and HER2-targeted TLR7/8 agonists, including BDC-1001 [176,177], supports the premise that the antibody serves as a localization device for an immune-active payload rather than for a cytotoxic one. The broader conceptual implication is that the ADC architecture is increasingly defined by its deliverable, with the antibody backbone treated as a configurable component within a broader mechanism-oriented therapeutic system [175]. The second trajectory is structural: bispecific and modular ADC platforms designed to address tumor heterogeneity at the design level rather than through bystander chemistry alone [178]. BL-B01D1, a bispecific ADC targeting both EGFR and HER3, has shown early signs of activity in heavily pretreated advanced solid malignancies, with a manageable safety profile, and serves as a proof of concept for the dual-antigen targeting strategy discussed in Section 7.1 [178].

**Figure 4:**
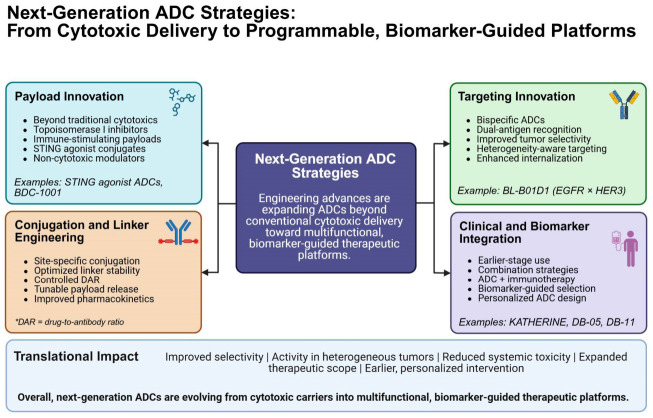
Next-generation antibody-drug conjugate strategies. This schematic outlines the key strategies in next-generation ADCs, grouped into four interrelated innovation areas: payload innovation, targeting innovation, engineering innovation, and clinical/biomarker integration. Payload innovations expand ADCs beyond cytotoxic agents to include topoisomerase I inhibitors, immune-activating payloads, and STING agonists. Targeting innovations include bispecific ADCs and dual-antigen recognition approaches. Engineering innovations include site-specific conjugation, improvements in linker stability, precise drug-to-antibody ratio (*DAR) control, payload release control, and pharmacokinetic optimization. Clinical applications involve utilization in early-stage disease, rational combinations, and biomarker-driven personalization.

The most significant near-term trajectory may be the transition from precision oncology to personalized ADC design. Current ADC selection rests on dichotomous antigen-expression criteria refined by quantitative thresholds (HER2-low, HER2-ultralow, FRα-high), but the field is shifting toward integrated decision frameworks that incorporate antigen density, spatial distribution, prior payload exposure, and serial biomarker dynamics [141,179]. Biomarker-driven analyses of T-DXd activity in DESTINY-CRC01 demonstrated the feasibility of combining tissue- and blood-based HER2 quantification to predict response [141]. Platforms such as ADC MATCH are operationalizing this concept prospectively by stratifying patients across HER2, Trop-2, and Nectin-4 expression tiers and assigning treatment accordingly [179]. The next-generation question for ADC selection is no longer whether a target is present but whether it is present at the right density, in the right spatial arrangement, in a patient whose prior therapy and resistance profile is compatible with the intended payload [141,142,179] (Figure 4).

## 12. Conclusions

Clinical efficacy of ADCs in solid tumors did not follow from improved antibody engineering. It followed from the linker-payload axis. The T-DXd versus T-DM1 comparison in DESTINY-Breast03 makes this concrete: an identical antibody backbone, differing only in linker–payload, drove the divergence in outcome [15]. The mechanistic explanation runs through bystander activity, which addresses the antigen heterogeneity that defeats internalization-dependent killing in mosaic tumors [16]. Sacituzumab govitecan extends the same logic, with extracellular payload release reducing dependence on conjugate internalization [18]. The three barriers mapped in Section 3 (vascular limitation, the binding-site barrier, and antigen mosaicism [1], the binding site barrier [7], and antigen mosaicism) prove addressable through chemistry without redesigning the targeting layer.

Clinical success, though, has reframed rather than resolved the field’s open questions. Resistance is not a single pathway but a coordinated failure across antigen modulation, trafficking, efflux, lysosomal processing, and apoptotic execution; each layer reinforces the others, and no predictive framework integrates them in individual tumors. Biomarker science has not caught up either. Immunohistochemistry remains the operative tool for patient selection despite its instability at HER2-low and HER2-ultralow thresholds [135,136], and ZEPHIR’s whole-body imaging data showed that tissue-defined HER2 status can disagree with lesion-level expression [150]. Liquid biopsy adds a temporal dimension but has not displaced tissue classification [141]. The sequencing problem deserves separate notice. As multiple ADCs converge on the same therapeutic space, the question of how prior exposure shapes subsequent response remains open [142], and the field has not produced trial evidence to settle it. Two trajectories define the platform’s near-term evolution. The first is stage migration. KATHERINE established that T-DM1 outperformed trastuzumab in residual HER2-positive early disease [98], and DESTINY-Breast05 and DESTINY-Breast11 have extended that logic to T-DXd [99,100]. ADCs are no longer tools for refractory metastatic disease alone; their evidentiary weight is moving forward in the treatment sequence. The second trajectory is payload diversification. Immune-stimulating constructs, bispecific architectures, and non-internalizing platforms are pushing the technology past cytotoxic delivery toward what reads more like mechanism-oriented therapy [174,175]. Whether these designs reach the same evidentiary level as T-DXd achieved in DESTINY-Breast03 is the open empirical question.

Section 10 examined whether the same delivery architecture transfers to autoimmune disease, and the answer remains genuinely undecided. Lineage-defining targets exist (CD19, CD20), pathway-driven targets exist (OX40, CD64), and GC-ADCs have cleared placebo-controlled efficacy endpoints [170,171]. What ABBV-154 also showed is that the autoimmune translation hurdle is not efficacy but differentiation. The comparator is a managed condition, the dosing horizon is years rather than months, and roughly 10–15% of RA patients meet the refractory definition that would justify a substantively riskier agent [172]. Anti-drug antibody emergence within 12 weeks (32.1% in ABBV-154 evaluable patients) leaves the chronic immunogenicity profile uncharacterized, and serious infection signals during placebo-controlled exposure track with what the broader biologic literature predicts [171,173]. Platform transferability is therefore an open question, not a settled one.

The next phase of ADC development depends less on new payload chemistries than on reconciling biomarker variability, resistance prediction, and sequencing logic across an increasingly crowded therapeutic space. The success of the linker-payload axis in solid tumors should not be read as a portable formula; the autoimmune extension is its own translational problem with distinct evidentiary requirements. The gaps flagged across this review (no integrated resistance framework, no validated tools for routine assessment of spatial and temporal target heterogeneity, no comparative evidence for ADC sequencing, no characterization of chronic immunogenicity in modulatory ADCs) define what the field has to deliver before the platform’s promise translates uniformly into patient benefit.

## Figures and Tables

**Table 1:** Approved antibody-drug conjugates (ADCs) for solid tumors.

ADC	Target	Tumor Type	Linker	Payload Class	Mechanism of Action	DAR	Key Trials	Median PFS/OS
Trastuzumab deruxtecan (T-DXd)	HER2	HER2+ and HER2-low/ultralow breast; HER2+ gastric; HER2-mutant NSCLC	Cleavable tetrapeptide (GGFG)	DXd (topoisomerase I inhibitor)	Topoisomerase I inhibition → DNA double-strand breaks; bystander effect	8	DESTINY-Breast03/04/06	PFS 29.0 vs. 7.2 mo (DB-03); PFS 10.1 vs. 5.4 mo (DB-04)
Trastuzumab emtansine (T-DM1)	HER2	HER2+ breast (metastatic and residual early disease)	Non-cleavable (SMCC)	DM1 (microtubule inhibitor)	Microtubule inhibition; non-cleavable, minimal bystander effect	3–4	EMILIA; KATHERINE	PFS 7.2 mo (vs. T-DXd, DB-03); iDFS benefit (KATHERINE)
Sacituzumab govitecan	Trop-2	Triple-negative and HR+/HER2− breast	Hydrolysable (CL2A)	SN-38 (topoisomerase I inhibitor)	Topoisomerase I inhibition; intracellular + extracellular payload release	7.6	ASCENT; TROPiCS-02	PFS 5.6 vs. 1.7 mo; OS 12.1 vs. 6.7 mo (ASCENT)
Datopotamab deruxtecan (Dato-DXd)	TROP2	NSCLC; HR+/HER2− breast	Cleavable tetrapeptide	DXd (topoisomerase I inhibitor)	Topoisomerase I inhibition; bystander effect	4	TROPION-Lung01; TROPION-Breast01	PFS 4.4 vs. 3.7 mo; HR 0.75 (Lung01)
Enfortumab vedotin (EV)	Nectin-4	Urothelial carcinoma	Cleavable (Val-Cit)	MMAE (microtubule inhibitor)	Microtubule inhibition	3.8	EV-301; EV-302 (+pembrolizumab)	OS 12.88 vs. 8.97 mo, HR 0.70 (EV-301); OS 31.5 vs. 16.1 mo (EV-302)
Patritumab deruxtecan (HER3-DXd)	HER3	EGFR-mutant NSCLC	Cleavable tetrapeptide	DXd (topoisomerase I inhibitor)	Topoisomerase I inhibition; bystander effect	8	HERTHENA-Lung01	ORR 30% (investigational)
Tisotumab vedotin	Tissue factor	Cervical cancer	Cleavable (Val-Cit)	MMAE (microtubule inhibitor)	Microtubule inhibition	4	innovaTV 204/301	OS 11.5 vs. 9.5 mo, HR 0.70 (cervical)
Mirvetuximab soravtansine	Folate receptor alpha (FRα)	FRα+ ovarian cancer	Cleavable (sulfo-SPDB)	DM4 (microtubule inhibitor)	Microtubule inhibition	3.5	SORAYA; MIRASOL	OS 16.46 vs. 12.75 mo, HR 0.67 (MIRASOL)
Telisotuzumab vedotin	c-MET	c-MET-high NSCLC	Cleavable (Val-Cit)	MMAE (microtubule inhibitor)	Microtubule inhibition	3.1	LUMINOSITY	ORR 35% (c-MET-high NSCLC, investigational)

DAR, drug-to-antibody ratio; PFS, progression-free survival; OS, overall survival; ORR, objective response rate; mo, months; NSCLC, non-small cell lung cancer. Values as reported in the cited pivotal trials.

**Table 2:** Summary of pivotal phase trials of ADCs in solid tumors.

Trial	Design/Phase	n	Key Efficacy Result	Hazard Ratio (HR)
DESTINY-Breast03	Randomized phase III, T-DXd vs. T-DM1	524	PFS 29.0 vs. 7.2 mo	0.28 (PFS)
DESTINY-Breast04	Randomized phase III, T-DXd vs. TPC (HER2-low)	557	PFS 10.1 vs. 5.4 mo; OS 23.9 vs. 17.5 mo	0.50 (PFS); 0.64 (OS)
DESTINY-Breast06	Randomized phase III, T-DXd vs. TPC (HER2-low/ultralow)	866	PFS 13.2 vs. 8.1 mo	0.63 (PFS)
ASCENT	Randomized phase III, sacituzumab govitecan vs. TPC (mTNBC)	468	PFS 5.6 vs 1.7 mo; OS 12.1 vs. 6.7 mo	0.41 (PFS); 0.48 (OS)
TROPiCS-02	Randomized phase III, sacituzumab govitecan vs. TPC (HR+/HER2−)	543	PFS 5.5 vs. 4.0 mo; OS 14.4 vs. 11.2 mo	0.66 (PFS); 0.79 (OS)
EV-301	Randomized phase III, enfortumab vedotin vs. chemo (urothelial)	608	OS 12.88 vs. 8.97 mo	0.70 (OS)
EV-302	Randomized phase III, EV + pembrolizumab vs. platinum chemo	886	PFS 12.5 vs. 6.3 mo; OS 31.5 vs. 16.1 mo	0.45 (PFS); 0.47 (OS)
TROPION-Lung01	Randomized phase III, Dato-DXd vs. docetaxel (NSCLC)	604	PFS 4.4 vs. 3.7 mo	0.75 (PFS)
MIRASOL	Randomized phase III, mirvetuximab soravtansine vs. chemo (FRα+ ovarian)	453	PFS 5.62 vs. 3.98 mo; OS 16.46 vs. 12.75 mo	0.66 (PFS); 0.67 (OS)
innovaTV 301	Randomized phase III, tisotumab vedotin vs. chemo (cervical)	502	OS 11.5 vs. 9.5 mo	0.70 (OS)

TPC, treatment of physician’s choice; mTNBC, metastatic triple-negative breast cancer; NSCLC, non-small cell lung cancer; n, number of randomized patients; mo, months. Patient numbers and outcomes as reported in primary trial publications; confirm against the manuscript’s reference list before submission.

## Data Availability

No new data were created or analyzed in this study. Data sharing is not applicable to this article.
